# Optogenetic control of YAP cellular localisation and function

**DOI:** 10.15252/embr.202154401

**Published:** 2022-07-25

**Authors:** Pearlyn J Y Toh, Jason K H Lai, Anke Hermann, Olivier Destaing, Michael P Sheetz, Marius Sudol, Timothy E Saunders

**Affiliations:** ^1^ Mechanobiology Institute National University of Singapore Singapore; ^2^ Department of Nephrology, Hypertension and Rheumatology University Hospital Münster Münster Germany; ^3^ Institute for Advanced Biosciences Université Grenoble Alpes Grenoble France; ^4^ INSERM U1209 Institute for Advanced Biosciences La Tronche France; ^5^ CNRS UMR 5039 Institute for Advanced Biosciences La Tronche France; ^6^ Department of Biochemistry and Molecular Biology University of Texas Medical Branch Galveston TX USA; ^7^ Icahn School of Medicine at Mount Sinai New York City NY USA; ^8^ Institute of Molecular and Cell Biology A*STAR Singapore; ^9^ Warwick Medical School University of Warwick Coventry UK

**Keywords:** cell proliferation, optogenetics, spatiotemporal dynamics, YAP, zebrafish, Methods & Resources, Signal Transduction

## Abstract

YAP, an effector of the Hippo signalling pathway, promotes organ growth and regeneration. Prolonged YAP activation results in uncontrolled proliferation and cancer. Therefore, exogenous regulation of YAP activity has potential translational applications. We present a versatile optogenetic construct (optoYAP) for manipulating YAP localisation, and consequently its activity and function. We attach a LOV2 domain that photocages a nuclear localisation signal (NLS) to the N‐terminus of YAP. In 488 nm light, the LOV2 domain unfolds, exposing the NLS, which shuttles optoYAP into the nucleus. Nuclear import of optoYAP is reversible and tuneable by light intensity. In cell culture, activated optoYAP promotes YAP target gene expression and cell proliferation. Similarly, optofYap can be used in zebrafish embryos to modulate target genes. We demonstrate that optoYAP can override a cell's response to substrate stiffness to generate anchorage‐independent growth. OptoYAP is functional in both cell culture and *in vivo*, providing a powerful tool to address basic research questions and therapeutic applications in regeneration and disease.

## Graphical abstract

optoYAP is a new optogenetic tool to manipulate the cellular localisation of YAP and YAP‐dependent gene expression. The efficacy and functionality of this tool is demonstrated in cell culture – including on different substrate stiffnesses – and zebrafish.

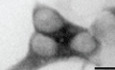

## Introduction

The Hippo signalling pathway regulates organ size control and cell fate during development and regeneration (Pan, 2007). Components of this pathway were identified in a *Drosophila melanogaster* genetic screen (Harvey *et al*, 2003; Huang *et al*, 2005) and are evolutionary conserved (Dong *et al*, 2007; Hilman & Gat, 2011). The core kinases in the Hippo pathway are MST1/2 and LATS1/2. MST1/2 phosphorylates and activates LATS1/2 (Chan *et al*, 2005) in the presence of SAV1 (Callus *et al*, 2006) and MOB1A/B (Hergovich *et al*, 2006). Subsequently, activated LATS1/2 phosphorylates YAP/TAZ (Huang *et al*, 2005) which results in their cytoplasmic sequestration from the nucleus by 14‐3‐3 proteins (Kanai *et al*, 2000; Basu *et al*, 2003) or their degradation (Zhao *et al*, 2010). Nuclear‐localised YAP/TAZ binds to TEAD transcription factors to mediate downstream gene expression (Zhao *et al*, 2008).

Cellular responses to Hippo signalling include proliferation, migration, changes in cellular cytoskeleton and morphology, as well as cell survival (Totaro *et al*, 2018). Genetic loss of the Hippo kinases or hyperactivation of YAP/TAZ (Yorkie in *D. melanogaster*) has been shown to promote tissue regeneration (Xin *et al*, 2013; Fu *et al*, 2017; Heallen *et al*, 2019), but at the risk of organ overgrowth (Wu *et al*, 2003; Dong *et al*, 2007; Morikawa *et al*, 2017). Furthermore, YAP/TAZ are also key drivers in tumour development (Zanconato *et al*, 2016). Therefore, a tool that can modulate YAP/TAZ function in a tightly controlled spatiotemporal manner is desirable.

Optogenetics is a powerful method for controlling the spatiotemporal localisation and activation of specific biological processes. It has been used extensively to activate neural responses (Yizhar *et al*, 2011). Optogenetic strategies have also been deployed to modulate and probe cell signalling pathways (Zhang & Cui, 2015) where high spatiotemporal control is desirable, such as Ras/Erk (Toettcher *et al*, 2013), Akt (Katsura *et al*, 2015), Notch (Viswanathan *et al*, 2019), Bicoid (Huang *et al*, 2017; Singh *et al*, 2022), Src (Kerjouan *et al*, 2021) and p53 signalling pathways (Niopek *et al*, 2016). Similarly, optogenetic tools have been applied in mechanobiology, studying junctional tension induced by RhoA (Cavanaugh *et al*, 2020) and talin‐mediated cell spreading (Yu *et al*, 2020). In the light of the variety of optogenetic tools and capabilities, optogenetic approaches potentially enable precise regulation of the YAP/TAZ nuclear‐cytoplasmic localisation, which can modulate their co‐transcriptional activity.

To this end, we utilised a previously developed tool LINuS, which contains the LOV2‐Jα interacting domain to photocage a NLS within the Jα helix domain (Niopek *et al*, 2014). Fusing this light‐inducible domain to the N‐terminus of YAP, henceforth referred to as optoYAP, thus enables manipulation of YAP cellular localisation with light, which we report here. We show that optoYAP is imported into the nucleus after a 488 nm light activation protocol in a range of cell lines and in the zebrafish embryo. We find that the optoYAP nuclear import dynamics are tuneable by activation light intensity. Activated optoYAP elicits cellular responses including the expression of downstream target genes and cell proliferation. Strikingly, we find that optoYAP can promote anchorage‐independent growth, showing that optoYAP activation is sufficient to override the mechano‐inhibitory signals in cells seeded on soft substrates. Such specific control opens new opportunities to explore key questions concerning the dysregulation of YAP localisation and its effects on disease progression.

## Results and Discussion

### Optogenetic YAP construct

**Figure 1:**
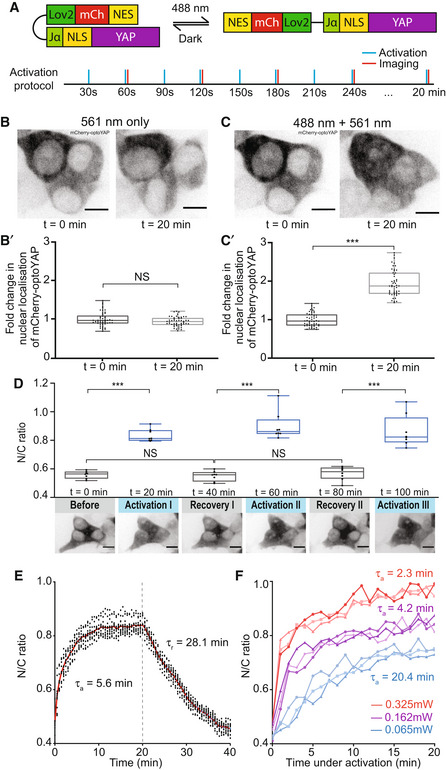
Characterisation of optoYAP in cell culture ASchematic of optoYAP construct. OptoYAP is folded in the dark due to the interaction between LOV2 domain and Jα helix. Under 488 nm light, nuclear localisation signal (NLS) is exposed and optoYAP is transported into the nucleus. Activation protocol was performed with 1 s blue light pulse every 30 s (blue bars), and cells were imaged every minute (red bars) for 20 min.BRepresentative images of mCherry‐optoYAP in HEK293T cells imaged with only 561 nm laser following the protocol in (A). Scale bars, 10 μm.B′Fold‐change in nuclear localisation of mCherry‐optoYAP (*n* = 50 cells from three independent experiments).CRepresentative images of the same cells in (B) exposed to both 488 and 561 nm following the protocol shown in (A). Scale bars, 10 μm.C′Fold‐change in nuclear localisation of the mCherry‐optoYAP (*n* = 50 cells from three independent experiments).DHEK293T cells transfected with optoYAP were subjected to three cycles of activation protocol and recovery in the dark. (Top) The ratio of mCherry‐optoYAP signal in the nucleus to the cytoplasmic mCherry‐optoYAP signal (*n* = 8 cells from two independent experiments). (Bottom) Representative images of the mCherry‐optoYAP signal from the same HEK293T cells at each cycle. Scale bars, 10 μm.EActivation time constant (τ_a_) and recovery time constant (τ_r_) (see Materials and Methods and Fig EV1B and C) of optoYAP in HEK293T cells. Vertical dashed line represents time when 488 nm stimulation ceased. Red line indicates average nuclear/cytoplasmic ratio (*n* = 12 cells).FHEK293T cells transfected with optoYAP subjected to 0.065, 0.163 and 0.325 mW 488 nm laser power at the focal plane. *N* = 3 cells per laser power. Data information: NS: Not significant, ***P < 10^−3^ (paired *t*‐test). Box plots represent median and 25^th^ to 75^th^ percentiles. Bars show minimum and maximum points.

To tightly control YAP localisation both temporally and spatially, we fused an optogenetic construct to the N‐terminal end of hYAP1 (Niopek *et al*, 2014). This construct consists of the LOV2 domain of *Avena sativa* phototropin 1 with mCherry for visualisation (Fig 1A). The LOV2 domain is connected to hYAP1 via a Jα helix and NLS sequence. A nuclear export signal (NES) derived from truncated cAMP‐dependent protein kinase (PKI) facilitates nuclear export and decreases background nuclear localisation in the dark state (Niopek *et al*, 2014). We refer to this construct as optoYAP (Fig 1A).

In the dark, the LOV2 domain photocages the NLS (Niopek *et al*, 2014) to prevent importation of optoYAP into the nucleus (Fig 1A). Under blue light illumination, the Jα helix domain unfolds and is released from the LOV2 domain to expose the NLS and allows transport of optoYAP into the nucleus.

### Characterisation of optogenetic YAP in mammalian cells

HEK293T cells were transiently transfected with optoYAP and subjected to 0.15 mW of 488 nm pulsatile illumination of 1 s every 30 s, similar to optogenetic activation protocols described previously (Niopek *et al*, 2014) (Fig 1A). Localisation of optoYAP is then visualised by the attached mCherry fluorophore. Whereas, optoYAP is localised to the cytoplasm without activation (Fig 1B and B'), its accumulation in the nucleus doubles after 20 min of activation (Fig 1C and C' and Movie EV1).

**Figure EV1:**
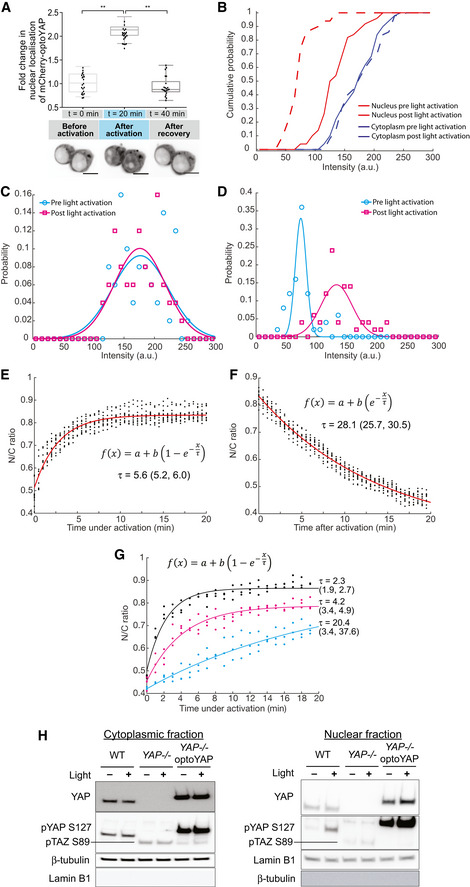
Characterisation of optoYAP in tissue culture cells AHEK293T transfected with optoYAP were subjected to activation protocol in Fig 1A followed by recovery in the dark for 20 min. Fold‐change in nuclear localisation of mCherry‐optoYAP (*n* = 22 cells from two independent experiments). Scale bars, 10 μm. Box plots represent median and 25^th^ to 75^th^ percentiles. Bars show minimum and maximum points, **P < 10^−2^ (paired *t*‐test).BCumulative distribution of nuclear and cytoplasmic intensity of 50 cells before and after light activation protocol. Data collection as described in Fig 1D.C, DHistogram of (C) cytoplasmic and (D) nuclear intensity of the same 50 cells, binned into 10 a.u. bins. Data collection as described in Fig 1D.E–GCurve fitting for Fig 1E. Red line represents the exponential curve fitted to the data. Numbers in brackets represent the 95% confidence interval of τ. (G) Curve fitting for Fig 1F for three different laser powers. Red line represents the exponential curve fitted to the data. Numbers in brackets represent the 95% confidence interval of τ. For (E–G), the given fitting parameters in the equations represent: *a* is the basal level of signal intensity; *b* is the multiplicative factor representing the change in signal due to light (de‐)activation; and τ represents the time scale over which the signal changes before/after light activation. Experimental data as described in Fig 1E and F.HWestern blots of MKN28 cells. MKN28 WT, *YAP* ^*−/−*^, and *YAP* ^*−/−*^ cells transfected with optoYAP were subjected to pulsed light activation for 48 h. Whole cell lysate from the three cell lines were separated into nuclear and cytoplasmic fractions, then probed for YAP, pYAP (S127), lamin B1 and β‐tubulin. Source data are available online for this figure.

We tested whether optoYAP shuttles in and out of the nucleus by recurring activation cycles. We subjected HEK293T cells to three rounds of activation protocol (20 min) and recovery in the dark (20 min). OptoYAP accumulates in the nucleus to the same level when activated and retreats to its baseline level after each activation cycle (Figs 1D and EV1A). We see a clear shift in the distribution of nuclear intensity post‐activation (Fig EV1B–D). The light intensity used (0.15 mW) is substantially lower than those reported to induce phototoxicity (Douthwright & Sluder, 2017) and we see no visible apoptosis in our analysed cells. Our results show that optoYAP activation is reversible and its light‐sensitive property is not attenuated over activation cycles.

To explore the nuclear import and export dynamics of optoYAP, we performed time‐lapse imaging of optoYAP in HEK293T cells. Cells were exposed to the above activation protocol for 20 min, and then the cells were left in the dark for a further 20 min, while optoYAP localisation was tracked every 30 s. The nuclear accumulation saturated quickly, with an activation time constant (τ_a_) of 5.6 ± 0.4 min (Figs 1E and EV1E and Materials and Methods). The recovery time constant (τ_r_) was 28.1 ± 2.4 min (Figs 1E and EV1F).

We next tested the light sensitivity of optoYAP in HEK293T cells. We performed a series of light activations using varying 488 nm laser power, 0.065, 0.162, and 0.325 mW, measured at the focal plane. Both τ_a_ (Fig EV1G) and extent of nuclear accumulation of optoYAP positively responded to increasing laser powers (Fig 1F).

Phosphorylation of YAP at serine 127 residue has been shown to promote its cytoplasmic localisation (Zhao *et al*, 2007). We investigated the phosphorylation status of optoYAP when it is nuclear localised after light activation. Using CRISPR‐mediated *YAP* knockout MKN28 cells, we transfected optoYAP into these cells and probed for pYAP (S127) using Western blot (Fig EV1H). Phosphorylated optoYAP was clearly present in the nucleus, suggesting that the NLS in our optoYAP construct can import phosphorylated optoYAP marked for cytoplasmic sequestration.

### Functionality of nuclear‐localised optoYAP

**Figure 2:**
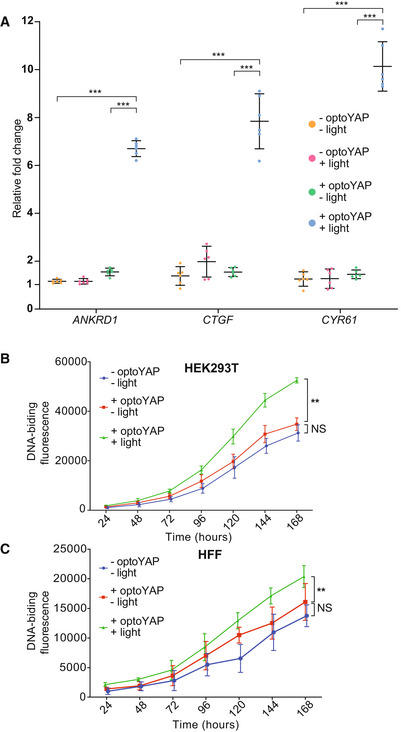
Light activation of optoYAP can activate downstream YAP target genes and control cell proliferation AExpression levels of *ANKRD1*, *CTGF*, and *CYR61* transcripts in HEK293T cells transfected with optoYAP after 48 h of activation protocol. Gene expression level was normalised to the housekeeping gene, *EIF1B*. Horizontal bars represent mean and 95% confidence interval from six biological replicates across two independent experiments for each condition, ***P < 10^−3^ (unpaired *t*‐test).B, CCell proliferation assay. HEK293T (B) and HFF (C) cells were transfected with optoYAP and subjected to activation protocol as described in Fig 1A or kept in the dark for 1 week. Error bars are s.d., *n* = 3 independent experiments for each condition, **P < 10^−2^ (unpaired *t*‐test).

**Figure EV2:**
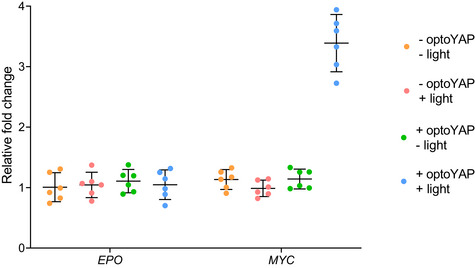
RT‐qPCR analysis of *EPO* and *MYC* HEK293T cells transfected with optoYAP were analysed for expression levels of *EPO* and *MYC* after 48 h of activation protocol. Erythropoietin (*EPO*) is a growth factor that is not induced by the Hippo‐YAP pathway and does not have an increase in transcript levels with activation of optoYAP. *MYC* is a target gene of YAP‐TEAD signalling and is increased in the presence of activated optoYAP. Gene expression was normalised to *EIF1B* housekeeping gene. Horizontal bars represent mean and 95% confidence interval from six biological replicates across three independent experiments for each condition.

We next investigated the functionality of optoYAP. As YAP is a transcriptional co‐regulator, it binds to TEAD transcription factors in the nucleus to initiate transcription of downstream genes in the context of the canonical Hippo pathway (Zhao *et al*, 2008). HEK293T cells transiently transfected with optoYAP were subjected to pulsed light activation over 48 h followed by qPCR assays of YAP target genes: *ANKRD1*, *CTGF* and *CYR61*. These genes are known downstream targets of TEAD‐mediated YAP activity (Zhao *et al*, 2008; Zhang *et al*, 2011; Stein *et al*, 2015). The expression levels of all three genes were significantly upregulated in cells with activated optoYAP construct as compared with control cells without light activation (Fig 2A). In addition, we analysed the expression level of erythropoietin (*EPO*) a growth factor that is not known to be induced by the Hippo‐YAP pathway (Singh *et al*, 2016). In this case, we saw no clear change in EPO after light activation of optoYAP, suggesting that optoYAP does not upregulate growth factors that are not TEAD‐mediated (Fig EV2). Although we did see some nuclear‐localised signal—especially in cells with high expression levels of optoYAP—the changes in target gene expression were stark and so low levels of nuclear localisation appear to not alter our conclusions. Furthermore, the changes seen in Fig 2A were clearly significant compared with untransfected cells under the same light conditions, suggesting that the light stimulation itself was not inducing the expression of these genes.

Given that activated optoYAP can activate downstream targets, we postulated that activated optoYAP could also promote cells to proliferate. We assayed cell proliferation of two cell lines, HEK293T and HFF, using a DNA‐binding fluorescent dye to measure their total DNA. Cells were transfected with optoYAP and subjected to pulsatile light activation over 1 week. Both transformed (HEK293T, Fig 2B) and non‐transformed (HFF, Fig 2C) cell lines show significant increase in total DNA with activated optoYAP as compared with untransfected and unactivated controls.

### optoYAP in zebrafish embryos

**Figure 3:**
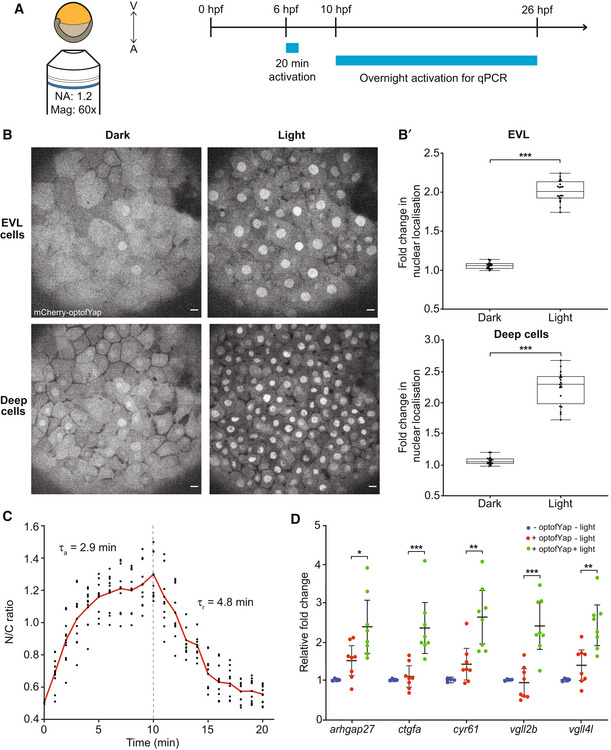
Validation of optofYap in zebrafish AImaging and light activation protocol in zebrafish. Shield‐stage embryos were subjected to activation protocol as described in Fig 1A and imaged at the animal pole.BExample images of embryos expressing optofYap (mCherry‐tagged) in the EVL and deep cells kept in the dark or subjected to the activation protocol at 6 hpf. Scale bars, 10 μm.B′Fold‐change in nuclear localisation of mCherry‐optofYap (*n* = 20 cells from three independent experiments). Box plots represent median and 25^th^ to 75^th^ percentiles. Whiskers show minimum and maximum points. ***P < 10^−3^ (paired *t*‐test).CThe N/C ratio measured using mCherry signal in zebrafish embryos injected with optofYap tracked over 10 min of activation protocol and 10 min recovery in the dark (see also Fig EV3A and B) (*n* = 10 cells from two independent experiments, red line = average). Vertical dashed line represents time when 488 nm stimulation ceased.DqPCR of Yap target genes. Embryos were kept in the dark until 10 hpf and then subjected to pulsed light activation for 16 h. Gene expression level was normalised to the housekeeping gene, *rpl13*. Horizontal bars represent mean and 95% confidence interval from four biological replicates for control and eight biological replicates for injected samples, *P < 0.05, **P < 10^−2^, ***P < 10^−3^ (unpaired *t*‐test).

**Figure EV3:**
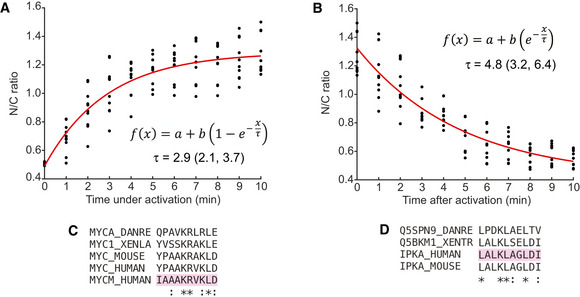
Characterisation of optofYap in zebrafish embryos A, BCurve fitting for Fig 3C. Red line represents the exponential curve fitted to the data. Numbers in brackets represent the 95% confidence interval of τ. For (A, B), the given fitting parameters in the equations represent: *a* is the basal level of signal intensity; *b* is the multiplicative factor representing the change in signal due to light (de‐)activation; and τ represents the time scale over which the signal changes before/after light activation. Experimental data as described in Fig 3C.C, DMultiple sequence alignments for the NLS of c‐Myc (C) and NES of PKI (D) between human, mouse, frog, zebrafish and the optogenetic construct backbone (highlighted in pink).

Building on the functional results in mammalian cell culture, we tested optoYAP in an *in vivo* system; the developing zebrafish embryo. We replaced hYAP by fYap; we refer to this construct as optofYap. Shield‐stage zebrafish embryos expressing *optofYap* mRNA were imaged at the animal pole (Fig 3A). optofYap is distributed uniformly in the entire cell prior to activation (Fig 3B). Using a 488 nm pulsatile light activation protocol as per Fig 1A, we see that optofYap can shuttle into the nuclei of both the enveloping layer (EVL) and the deep cells (Fig 3B and Movie EV2). We observe a twofold change in nuclear localisation after light activation (Fig 3B'). Interestingly, optofYap appears to enter and leave the nucleus much faster in the zebrafish embryos than optoYAP does in cultured cells. The activation time constant was calculated to be 2.9 ± 0.8 min and recovery time constant at 4.8 ± 1.6 min (Figs 3C and EV3A and B).

Similar to our tissue culture studies, we tested whether optofYap can induce the expression of specific Yap target genes in the embryo after activation (Kimelman *et al*, 2017). qPCR was performed on embryos subjected to overnight activation protocol for 14 h from the tailbud stage (10 h post fertilisation (hpf)). The expression of all downstream genes was upregulated after activation (Fig 3D). This shows that optofYap can induce upregulation of target genes *in vivo*.

### optoYAP can induce anchorage‐independent growth

**Figure 4:**
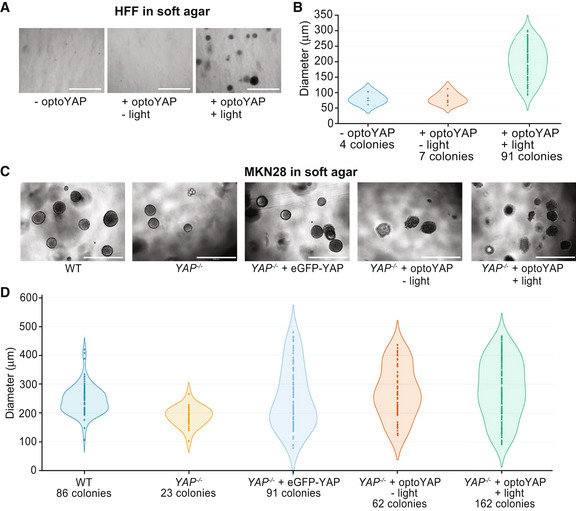
Functional assays of optoYAP in tissue culture cells A–DSoft agar colony formation assay for HFF (A, B) and MKN28 (C, D) cells respectively grown on soft agar for 1 week under different light conditions as described in Fig 1A. Scale bars, 1 mm. (A, C) Representative images of colony growth in HFF and MKN28 cells, respectively, under different activation conditions. (B, D) Diameter of individual colonies formed under different light conditions and genetic backgrounds. Colonies were counted and measured from three biological replicates.

**Figure EV4:**
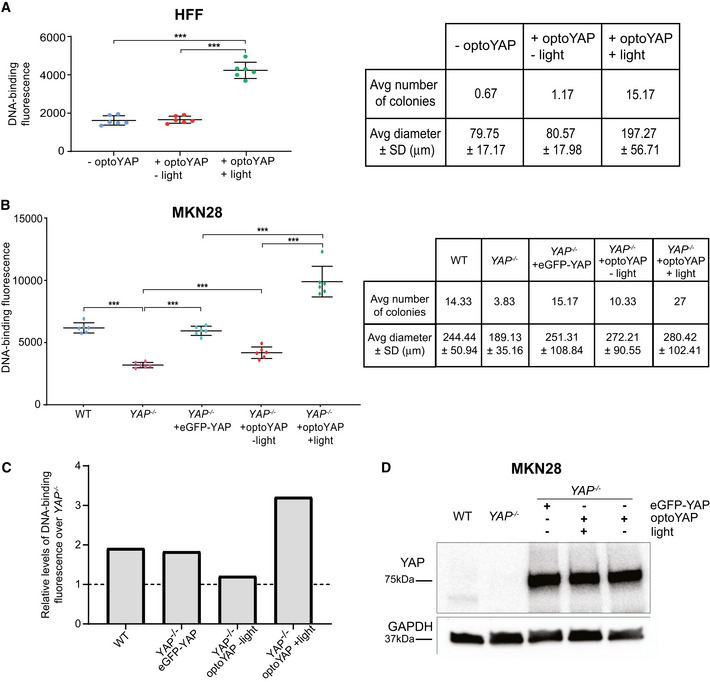
Colony formation assay A, BQuantification of DNA‐binding fluorescent dye in HFF (A) and MKN28 (B) cells grown on soft agar. The average number and diameter of colonies formed (representative images in Fig 4A and C) are shown in the table on the right. Error bars are s.d., *n* = 6 biological replicates from two independent experiments for each condition, ***P < 10^−3^ (unpaired *t*‐test).CRelative levels of DNA‐binding fluorescence as compared to *YAP* ^*−/−*^ by dividing the average fluorescence level of each condition in (B) by the average fluorescence of *YAP* ^*−/−*^.DWestern blots of different MKN28 cell lines. eGFP‐YAP and optoYAP lines are transfected into the *YAP* ^*−/−*^ background.

Mechanical parameters of the microenvironment, such as matrix rigidity, are crucial for normal cell growth (Wang *et al*, 2000). In particular, epithelial cells typically cannot survive and proliferate on soft substrates. However, transformed cells are able to bypass rigidity sensing and grow on soft substrates (Yang *et al*, 2020). We hypothesised that activated optoYAP may drive proliferation of cells even on soft substrates. Using a non‐transformed cell line, HFF, we cultured untransfected and optoYAP‐transfected cells with or without light activation on soft agar for 7 days. As expected, few untransfected cells survived, with little visible colony formation (Fig 4A). However, optoYAP‐transfected cells that were subjected to light activation formed more colonies (Figs 4A and EV4A) with larger size (Fig 4B) compared to those without light activation. The increase in number and size of colonies formed indicates that activated optoYAP can induce transformed growth, overriding the ability of normal cells to sense their microenvironment and suppress growth in soft substrates.

Similarly, we performed the soft agar assay on a transformed cell line MKN28. Using MKN28 CRISPR *YAP* ^*−/−*^ cells (Qiao *et al*, 2017), we rescued these mutant cells with either eGFP‐YAP or optoYAP. We seeded four variants of MKN28 cells—wild‐type (WT), *YAP* ^*−/−*^, *YAP* ^*−/−*^ + eGFP‐YAP and *YAP* ^*−/−*^ + optoYAP—on soft agar for 7 days. WT MKN28 cells, unlike HFF cells, are transformed cells and are able to form colonies on soft agar, which is attenuated by the loss of *YAP* (Figs 4C and D, and EV4B). While overexpression of eGFP‐YAP rescues *YAP* null MKN28 cells to form colonies at WT levels, activated optoYAP exaggerates colony formation of MKN28 cells on soft agar (Figs 4C and EV4C). Protein expression levels of both eGFP‐YAP and optoYAP transfected in *YAP* null cells were similar (Fig EV4D). However, optoYAP was localised in the nucleus during the activation protocol, therefore likely inducing cell proliferation and increasing the number of colonies formed (Figs 4D and EV4B). The colony size did not vary between the different MKN28 cell lines, with the exception of *YAP* null cells, but the number of colonies formed with activated optoYAP is double of the WT or eGFP‐YAP rescue (Fig EV4B). These data show that exogenous and sustained activation of optoYAP in MKN28 cells triggers greater levels of cell growth on soft agar than that of WT or rescued *YAP* mutant cells. This suggests that optoYAP activation alone is sufficient to promote colony formation on soft substrates in both normal and transformed cells.

Here, we report the development of a functional optogenetic YAP, optoYAP, by exploiting the light‐responsive LOV2‐Jα domain to photocage NLS. We characterised optoYAP function in human cells and zebrafish embryos, showing a consistent increase in YAP nuclear localisation upon blue light illumination within minutes. This rapid manipulation of YAP localisation elicits downstream responses, including the upregulation of target genes, increased cell proliferation in cell culture and anchorage‐independent growth. Long‐term activation of optoYAP does not appear to have detrimental effects in cell culture. Each activation cycle is independent (Fig 1D), and cells survive after being subjected to 1 week of light activation. This adds to the advantage of optogenetic YAP over constitutively active YAP or drug‐induced YAP expression.

Classical genetic approaches to study signalling pathways such as overexpression or knockouts/knockdowns have comparatively lesser control over spatial and temporal variation (Moya & Halder, 2019). Other approaches such as drug/ligand‐mediated expression can also be difficult to precisely control due to binding kinetics and diffusion of small molecules in tissues. On the contrary, optogenetics present a robust and predictable activation mechanics. Light activation can be easily and quickly modulated in space, time and intensity, allowing for detailed manipulation of the protein of interest (Repina *et al*, 2017), providing a more powerful tool for manipulating YAP than previous approaches.

A similar optogenetic YAP based on the LOV2‐TRAP tool has recently been described (Dowbaj *et al*, 2021). However, the construct does not show a functional YAP response upon activation. There, LOV2 is tethered on the outer surface of the mitochondria and YAP is tagged with a small peptide Zdk which interacts with the LOV2 domain (Dowbaj *et al*, 2021) and is suited to study the protein dynamics of nuclear import and export by sequestering YAP to the mitochondria. This system requires two plasmids to be introduced into the tissue of interest. Another approach (Illes *et al*, 2021) has taken advantage of a light‐activated NLS construct (Engelke *et al*, 2014) to control YAP localisation to the nucleus. This approach could stimulate growth of cancer spheroids, but it was irreversible (at least on hour time scales) and the time scales for activation were typically over 30 min. Our optogenetic YAP construct described here requires only a single component, the LOV2‐Jα interacting domain, thus simplifying its deployment and presents an advantage for future applications. Activation of the photosensitive domain is rapid (minute time scales) and can be achieved within the visible light range at 488 nm and recovery done in the dark. These features add to the ease of use over other optogenetic approaches that require multiple components, activation in the infrared spectrum or are slower and irreversible (Tischer & Weiner, 2014). To the best of our knowledge, our work reports the first functional optogenetic YAP construct. This system allows for robust spatiotemporal control of YAP activity, enabling the understanding of how YAP can be manipulated to induce downstream phenotypic changes.

We observed two differences in our optogenetic construct between tissue culture cells and zebrafish embryos. First, without activation, similar levels of optofYap in the nucleus and cytoplasm were observed in zebrafish embryos (Fig 3B), in contrast to optoYAP which is localised to the cytoplasm of human cell lines (Fig 1C). Second, the activation time constant is faster in zebrafish (2.9 min, Fig 3C) than in cultured cells (5.6 min, Fig 1E). The NLS and NES present in this optogenetic construct were optimised from bipartite NLS of nucleoplasmin and human interleukin‐5, and PKI, respectively (Niopek *et al*, 2014). These two peptide sequences differ from their respective *Danio rerio* orthologue (Fig EV3C and D). This sequence divergence could be an explanation for the differing dynamics of optoYAP between human and zebrafish cells. The basal rate of nuclear export can be interpreted to be lower in zebrafish embryos as compared with tissue culture cells, as seen in the distribution of optogenetic YAP in the dark state (Figs 1B and 3B). In cell culture, there is a clear nuclear exclusion of optoYAP in the dark (Fig 1B). The nuclear import and export rates also differ in cell culture (Fig 1E), which suggests that here the rate of nuclear import driven by the unmasked NLS is greater than the rate of nuclear export driven by the NES (which is dependent on the exportins present in the different systems). The specific reasons for these differences are unclear—*for example* the comparative strength of NLS and NES in human and zebrafish cells—and is for future study.

Using non‐transformed human cells, HFF, we showed a substantial increase in the number and size of colonies formed by the activated optoYAP that induced transformed growth, overriding the ability of normal cells to sense their microenvironment and suppress growth in soft substrates (Fig 4A and B). Often transformed cell lines have several mutated oncogenes and/or tumour suppressors, which act in concert, to elicit a clear transformation phenotype (Hanahan & Weinberg, 2011). In our hands, forcing a single, non‐mutated optoYAP proto‐oncogene protein to the cell nucleus by light was sufficient for the cells to display one of the critical hallmarks of neoplastic transformation, anchorage‐independent growth.

We found that a transformed cell line, MKN28, depends on YAP for anchorage‐independent growth, as the removal of *YAP* results in fewer and smaller colonies in the soft agar assay. Moreover, forced overexpression of eGFP‐YAP in these mutant cells reinstated colony growth similar to WT cell levels. On the contrary, light activation of optoYAP further amplified cell proliferation by increasing the number of colonies formed. One possibility is that eGFP‐YAP is limited by upstream signals including Hippo kinases, and that our optoYAP appears to override such limitations as seen in our Western blot data (Fig EV1E). This shows that optoYAP might be able to bypass canonical Hippo signalling regulation, making it a potentially powerful tool for manipulating growth *in vivo*.

YAP signalling has been shown to affect cell fate by transducing mechanical signals from the extracellular environment (Hao *et al*, 2014; Totaro *et al*, 2017). In both qPCR data from tissue culture cells (Fig 2A) and zebrafish embryos (Fig 3D), two key genes—*ctgfa/b* and *cyr61*—are upregulated and these play important roles in mediating tissue remodelling. CTGF modulates cell adhesion and migration, and extracellular matrix deposition and remodelling (Shi‐Wen *et al*, 2008; Lipson *et al*, 2012). CYR61 mediates cell survival, differentiation and proliferation (Lau, 2011). When activated, CYR61 has been shown to promote wound healing in human skin fibroblasts and endothelial cells by interacting with integrin receptors to induce cell migration (Chen *et al*, 2001). Both genes are significantly upregulated upon optoYAP activation in cell culture and in zebrafish, which shows that optoYAP signalling is likely able to affect cell fate through activation of *ctgf* and *cyr61*. We note that while the observed change in target gene activation in cell culture was comparable with other approaches to enhance YAP activity (Hao *et al*, 2014), the fold change in the early embryo was comparatively small. This is likely due to tissue‐specific differences and the relatively short time scale for gene activation compared with cell culture or *in vivo* work in adults (Hao *et al*, 2014). This approach should be extendable to longer‐term experiments (such as manipulating organ growth and repair after wounding), although care is needed to ensure robust optogenetic activation (Toettcher *et al*, 2011).

Overall, given the importance of YAP with respect to development and regeneration, and the ease of the optogenetic tool presented here, optoYAP has the potential to become a powerful tool for studying Hippo‐YAP signalling in both research and clinical applications.

## Materials and Methods

### Plasmid construction

Optogenetic plasmid (pDN34, Addgene plasmid #61343, previously reported in (Niopek *et al*, 2014)) containing the LOV2‐Jɑ domain was a gift from Barbara Di Ventura and Roland Eils. Human YAP1‐1δ was PCR amplified from pcDNA3.1 hYAP1‐1δ with primers AH05 and AH06 (listed in Table EV1). Phusion High Fidelity DNA polymerase (ThermoScientific) was used for PCR amplification. PCR product and pDN34 were cleaved with KpnI and PmeI FastDigest restriction enzymes (ThermoScientific) for cloning. Optogenetic backbone fused to hYAP1‐1δ is referred to as optoYAP.

### Mammalian cell culture and transfection

HEK293T and HFF cells were kept in phenol red‐free Dulbecco's modified Eagle medium (DMEM) (Gibco) supplemented with 10% (V/V) heat‐inactivated foetal bovine serum (FBS) (Hyclone) and 1% (V/V) penicillin/streptomycin (Nacalai Tesque). MKN28 cells were kept in RPMI 1640 supplemented with 10% (V/V) heat‐inactivated FBS (Hyclone) and 1% (V/V) penicillin/streptomycin (Nacalai Tesque). All cells were maintained in 37°C incubator with 5% CO_2_.

HEK293T and HFF cells were transfected with Lipofectamine 2000 (Invitrogen) according to the manufacturer's instructions. MKN28 cells were transfected using K2 transfection system (Biontex).

### Zebrafish strains

All zebrafish strains were maintained according to standard fish husbandry procedures. The AB wild‐type strain was used in this study. All experiments with zebrafish were approved by the A*STAR Biological Resource Centre according to the Singapore National Advisory Committee on Laboratory Animal Research.

### Zebrafish microinjection

*fYap* was cloned from pCS2 *eGFP‐fYap* into the optogenetic pDN34 backbone. The entire construct containing the optogenetic domains and *fYap* was then cloned into a pCS2 vector backbone by restriction enzyme cloning with EcoRI and PspXI (ThermoScientific) from optoYAP. The plasmid was linearised using NotI (ThermoScientific) and capped mRNA was synthesised using mMessage Machine SP6 kit (Ambion). Embryos from AB zebrafish were collected at one‐cell stage and injected with 50 pg *optofYap* mRNA.

### Fluorescence microscopy

Cells were visualised under the microscope about 24 h post transfection. Live imaging was performed on cells seeded on Iwaki glass‐bottom dish and imaged on Perkin Elmer spinning disc with a LUCPlanFLN 40x/0.6 NA air objective. Injected zebrafish embryos were collected about 6 h post fertilisation at shield stage, dechorionated and embedded in 1% low melting agarose dissolved in egg media. Embryos were mounted on a MatTek dish and imaged on Nikon Ti‐E with Yokogawa W1 spinning disc with a PLAN APO VC 60X/1.20 NA water immersion objective.

The nuclear/cytoplasmic ratio was measured using Fiji by drawing a region of interest (ROI) around the cell and its nucleus. A macro plugin was used to mask the nucleus from the cytoplasm to calculate the mean intensity in the nucleus and cytoplasm based on the ROI drawn. Nucleus was demarcated by region without mCherry signal in cells at *t* = 0 min and region with highest level of mCherry in zebrafish embryos at *t* = 20 min. Laser power at the focal point was measured using a power meter (Thorlabs).

### Light activation

Pulsed light activation is as follows: cells are illuminated with a 1 s pulse of 0.15 mW 488 nm laser light, followed by a 30 s dark phase. This pulsation is continued over 20 min on the Perkin Elmer or Nikon W1. For long‐term cell proliferation (7 d), soft agar assay (7 d) and qPCR experiments (48 h for tissue culture cells, 16 h for zebrafish embryos), cells were kept in cell culture incubators fitted with a LED light strip that pulsed at the same frequency.

### RT‐qPCR

Total RNA was isolated from mammalian cell culture after 48 h of pulsatile light activation using RNeasy Mini Kit (Qiagen) according to the manufacturer's instructions. Complementary DNA (cDNA) was synthesised from isolated RNA using SuperScript IV Reverse Transcriptase (ThermoScientific). Zebrafish embryos injected with optofYap at the one‐cell stage were subjected to pulsatile light activation and collected after 48 h.

For zebrafish, five embryos were pooled into one biological replicate and a total of four biological replicates were obtained for each sample. RNA was extracted using TRIzol (Ambion) and purified with Direct‐zol RNA kit (Zymogen). cDNA was synthesised using High‐Capacity RNA‐to‐cDNA kit (ThermoScientific).

Real‐time PCR detection for both cell culture samples and zebrafish embryos were made using SYBR Green PCR Master Mix for 40 cycles in a Bio‐Rad CFX96 thermal cycler. The threshold cycle (Ct) value for each gene was normalised to the Ct value of a housekeeping gene, *EIF1B* and *rpl13* for cell culture and zebrafish, respectively. The relative fold changes were calculated using ΔΔCt method. The primer sequences for target genes are listed in Table EV1.

### Soft agar assay

Soft agar assay was performed using CytoSelect 96‐Well Cell Transformation Assay (Cell Biolabs) according to the manufacturer's instructions. Cells were grown in T25 flasks to 80% confluency, trypsinised and counted with a haemocytometer to obtain the concentration of live cells. Based on these measures, we seeded each well with 5,000 cells. The final 0.4% agar layer on which cells were grown in corresponds to a Young's modulus of < 2 kPa.(Li *et al*, 2011) 7 days after seeding, cell colony formation was examined with EVOS Cell Imaging System (ThermoScientific) under 10× magnification. Cell proliferation was measured with CyQuant NF Cell Proliferation Assay Kit (ThermoScientific) according to the manufacturer's instructions.

### Cell proliferation assay

Cells were grown in T25 flasks to 80% confluency, trypsinised and counted with a haemocytometer. Cells were plated in 96‐well plates at 1,000 cells per well and fluorescent intensity was measured every 24 h post seeding for 7 days according to CyQuant NF Cell Proliferation Assay (ThermoScientific) for adherent cells.

### Western blotting

Cells were seeded in six‐well plates at 300,000 cells per well, and cell lysate was harvested 48 h post seeding. Nuclear and cytoplasmic fractions were separated using NE‐PER Nuclear and Cytoplasmic Extraction Reagents (ThermoScientific) according to the manufacturer's instructions for adherent cells. Protein concentration of both fractions were measured using Pierce BCA Protein Assay Kit (ThermoScientific). 15 μg of protein was separated by 4–20% Mini Protean TGX SDS‐polyacrylamide gels (Bio‐Rad) at 120 V for 1 h and transferred onto 0.2 μm PVDF membranes (Bio‐Rad) at 90 V for 1 h. Membranes were blocked with 3% (W/V) BSA in TBST for 1 h at room temperature (RT) and incubated overnight at 4°C with primary antibodies in 3% (W/V) BSA in TBST. Membranes were washed three times for 5 min with TBST and incubated with either anti‐mouse or rabbit HRP‐conjugated secondary antibodies in 3% (W/V) BSA in TBST for 1 h at RT. Membranes were washed three times for 5 min with TBST after secondary antibody incubation and chemiluminescence signal was detected using Clarity Western ECL Substrate (Bio‐Rad). All relevant information on antibodies used in this study are listed in Table EV2.

### Statistical analysis

Biological replicates refer to independent experimental replicates with independent transfections, sample preparations and measurements on different days.

### Curve fitting

Time constant (τ) of optoYAP import and export from the nucleus was fitted using the curve fitting function *fit* in MATLAB. Nuclear import rate (τ_a_) was fitted with $f\left(x\right)=a+b\left(1-e^{-\frac{x}{\tau }}\right)$ and export rate (τ_r_) was fitted with $f\left(x\right)=a+b\left(e^{-\frac{x}{\tau }}\right)$.

## Author contributions

**Pearlyn J Y Toh:** Data curation; formal analysis; validation; investigation; visualization; methodology; writing – original draft; writing – review and editing. **Jason K H Lai:** Data curation; supervision; investigation; writing – review and editing. **Anke Hermann:** Resources. **Olivier Destaing:** Resources. **Michael P Sheetz:** Resources; supervision; funding acquisition. **Marius Sudol:** Conceptualization; resources; supervision; funding acquisition; writing – review and editing. **Timothy E Saunders:** Conceptualization; resources; supervision; funding acquisition; project administration; writing – review and editing.

In addition to the CRediT author contributions listed above, the contributions in detail are:

PJYT, MS and MPS conceived the study. PJYT, JKHL, MS, MPS, TES designed and planned the experiments. PJYT performed the cell culture and zebrafish experiments. JKHL assisted with the experiments, particularly in the zebrafish work. AH generated the initial optoYAP clone with the help of OD. PJYT analysed the data with assistance from JKHL and TES. PJYT, JKHL and TES wrote the first draft of the manuscript with all authors approving the final manuscript.

## Disclosure and competing interests statement

The authors declare that they have no conflict of interest.

## Supporting information



Expanded View Figures PDF

Table EV1

Table EV2

Movie EV1

Movie EV2

Source Data for Expanded view

## Data Availability

No data have been generated that require deposition in a public database. All data in this study are available upon request.
