# Supplementary material for: Optogenetic control of YAP cellular localisation and function
Source: EMBO Rep. 2022 Jul 25;23(9):e54401. doi: 10.15252/embr.202154401 (PMC9442306; doi:10.15252/embr.202154401)
Supplement: Supplementary file 1 — Expanded View Figures PDF [file EMBR-23-e54401-s001.pdf]

Expanded View Figures

Figure EV1. Characterisation of optoYAP in tissue culture cells.

A HEK293T transfected with optoYAP were subjected to activation protocol in Fig 1A followed by recovery in the dark for 20 min. Fold-change in nuclear localisation of mCherry-optoYAP ($n = 22$ cells from two independent experiments). Scale bars, 10 μm. Box plots represent median and 25th to 75th percentiles. Bars show minimum and maximum points, $^{**}P < 10^{-2}$ (paired t-test).

B Cumulative distribution of nuclear and cytoplasmic intensity of 50 cells before and after light activation protocol. Data collection as described in Fig 1D.

C, D Histogram of (C) cytoplasmic and (D) nuclear intensity of the same 50 cells, binned into 10 a.u. bins. Data collection as described in Fig 1D.

E–G Curve fitting for Fig 1E. Red line represents the exponential curve fitted to the data. Numbers in brackets represent the 95% confidence interval of τ. (G) Curve fitting for Fig 1F for three different laser powers. Red line represents the exponential curve fitted to the data. Numbers in brackets represent the 95% confidence interval of τ. For (E–G), the given fitting parameters in the equations represent: a is the basal level of signal intensity; b is the multiplicative factor representing the change in signal due to light (de-)activation; and τ represents the time scale over which the signal changes before/after light activation. Experimental data as described in Fig 1E and F.

H Western blots of MKN28 cells. MKN28 WT, *YAP*$^{-/-}$, and *YAP*$^{-/-}$ cells transfected with optoYAP were subjected to pulsed light activation for 48 h. Whole cell lysate from the three cell lines were separated into nuclear and cytoplasmic fractions, then probed for YAP, pYAP (S127), lamin B1 and β-tubulin.

Source data are available online for this figure.

© 2022 The Authors

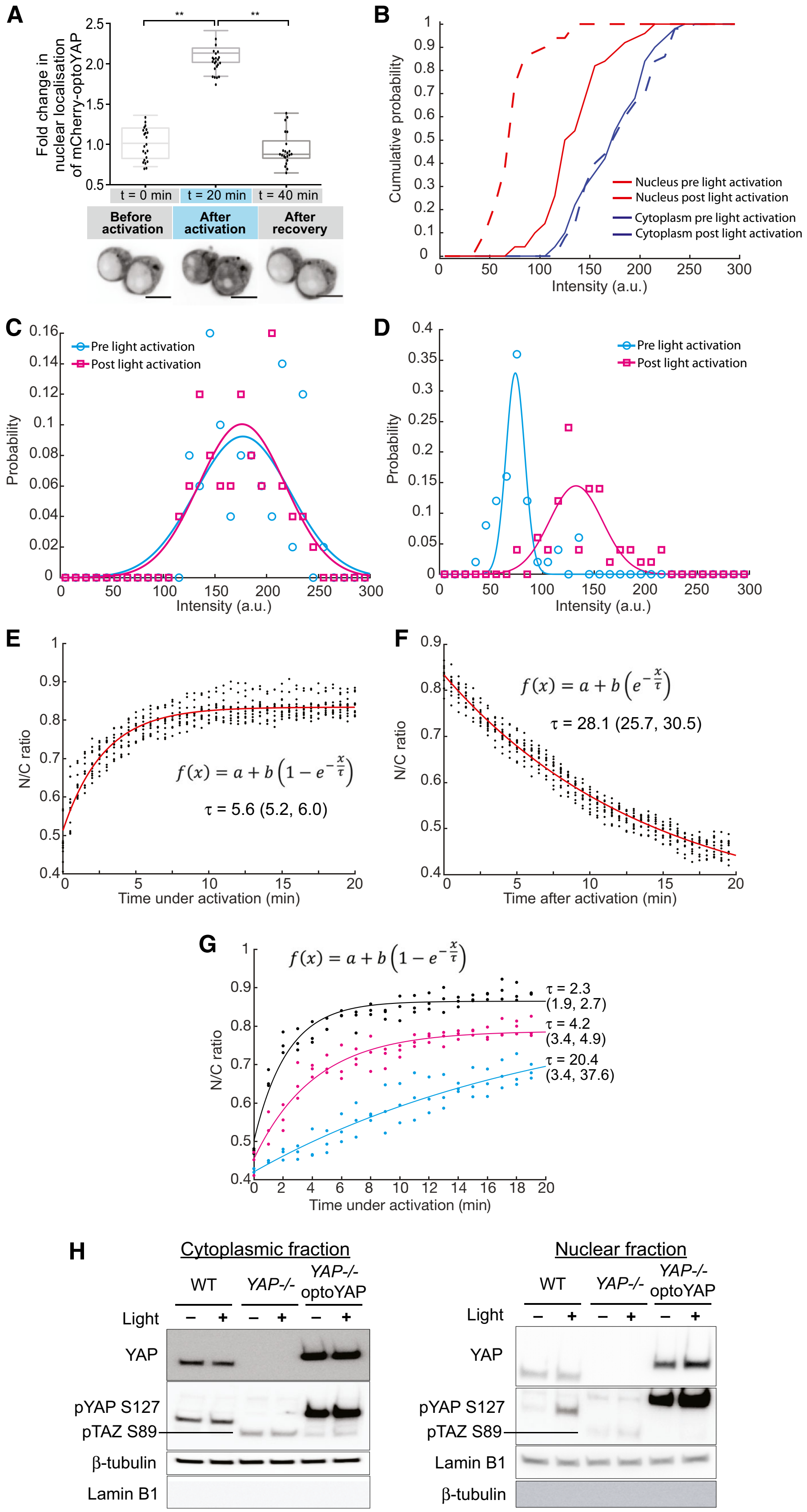
A
Fold change in nuclear localisation of mCherry-optoYAP
t = 0 min
t = 20 min
t = 40 min
Before activation
After activation
After recovery
B
Cumulative probability
Intensity (a.u.)
Nucleus pre light activation
Nucleus post light activation
Cytoplasm pre light activation
Cytoplasm post light activation
C
Probability
Pre light activation
Post light activation
Intensity (a.u.)
D
Probability
Pre light activation
Post light activation
Intensity (a.u.)
E
N/C ratio
$f(x) = a + b\left(1 - e^{-\frac{x}{\tau}}\right)$
τ = 5.6 (5.2, 6.0)
Time under activation (min)
F
N/C ratio
$f(x) = a + b\left(e^{-\frac{x}{\tau}}\right)$
τ = 28.1 (25.7, 30.5)
Time after activation (min)
G
N/C ratio
$f(x) = a + b\left(1 - e^{-\frac{x}{\tau}}\right)$
τ = 2.3 (1.9, 2.7)
τ = 4.2 (3.4, 4.9)
τ = 20.4 (3.4, 37.6)
Time under activation (min)
H
Cytoplasmic fraction
Nuclear fraction
WT
YAP-/-
YAP-/- optoYAP
Light
YAP
pYAP S127
pTAZ S89
β-tubulin
Lamin B1

Figure EV1.

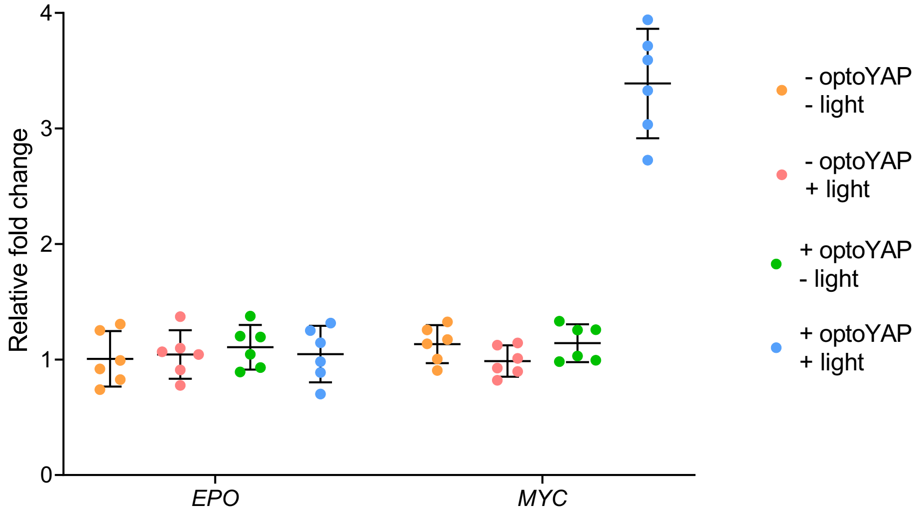

Figure EV2. RT-qPCR analysis of *EPO* and *MYC*.

HEK293T cells transfected with optoYAP were analysed for expression levels of *EPO* and *MYC* after 48 h of activation protocol. Erythropoietin (*EPO*) is a growth factor that is not induced by the Hippo-YAP pathway and does not have an increase in transcript levels with activation of optoYAP. *MYC* is a target gene of YAP-TEAD signalling and is increased in the presence of activated optoYAP. Gene expression was normalised to *EIF1B* housekeeping gene. Horizontal bars represent mean and 95% confidence interval from six biological replicates across three independent experiments for each condition.

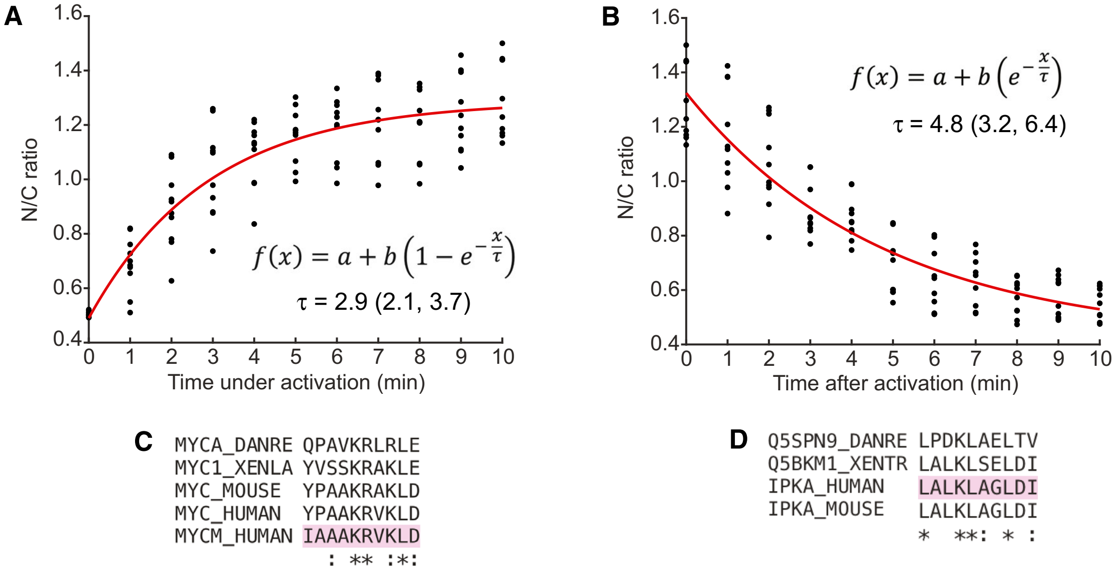

Figure EV3. Characterisation of optofYap in zebrafish embryos.

A, B Curve fitting for Fig 3C. Red line represents the exponential curve fitted to the data. Numbers in brackets represent the 95% confidence interval of τ. For (A, B), the given fitting parameters in the equations represent: a is the basal level of signal intensity; b is the multiplicative factor representing the change in signal due to light (de-)activation; and τ represents the time scale over which the signal changes before/after light activation. Experimental data as described in Fig 3C.

C, D Multiple sequence alignments for the NLS of c-Myc (C) and NES of PKI (D) between human, mouse, frog, zebrafish and the optogenetic construct backbone (highlighted in pink).

© 2022 The Authors

A

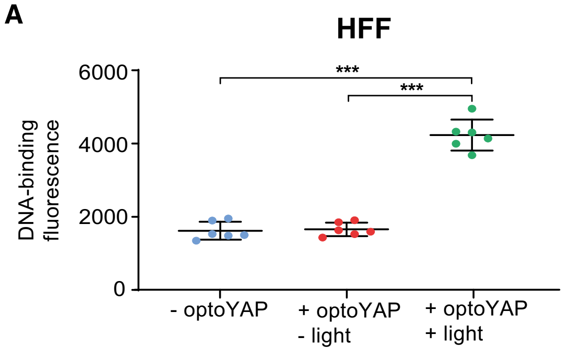

	- optoYAP	+ optoYAP - light	+ optoYAP + light
Avg number of colonies	0.67	1.17	15.17
Avg diameter ± SD (μm)	79.75 ± 17.17	80.57 ± 17.98	197.27 ± 56.71

B

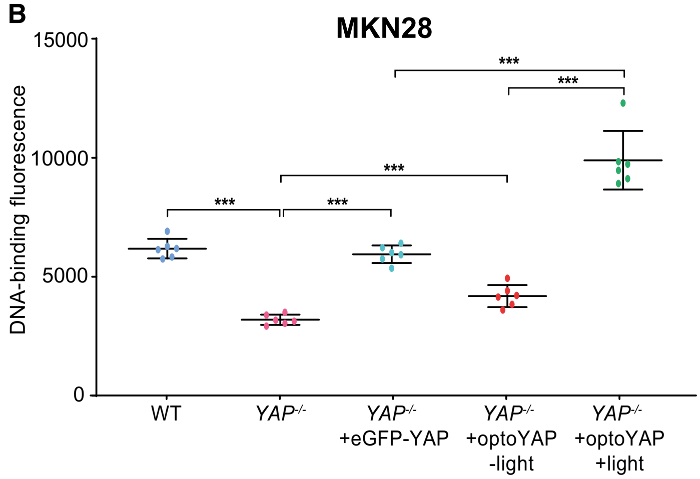

	WT	*YAP*-/-	*YAP*-/- +eGFP-YAP	*YAP*-/- +optoYAP - light	*YAP*-/- +optoYAP + light
Avg number of colonies	14.33	3.83	15.17	10.33	27
Avg diameter ± SD (μm)	244.44 ± 50.94	189.13 ± 35.16	251.31 ± 108.84	272.21 ± 90.55	280.42 ± 102.41

C D

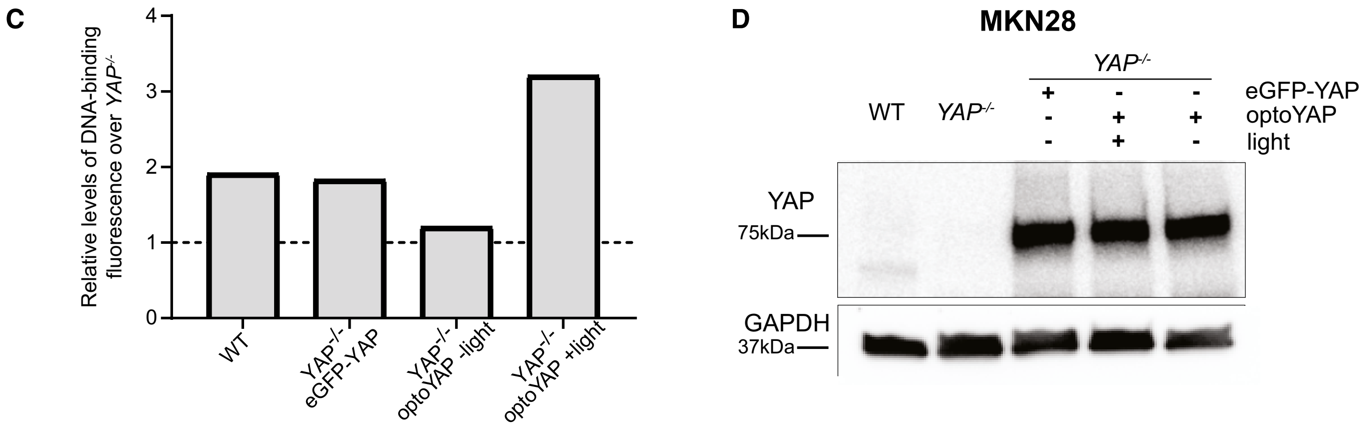

Figure EV4. Colony formation assay.

A, B Quantification of DNA-binding fluorescent dye in HFF (A) and MKN28 (B) cells grown on soft agar. The average number and diameter of colonies formed (representative images in Fig 4A and C) are shown in the table on the right. Error bars are s.d., $n = 6$ biological replicates from two independent experiments for each condition, $***P < 10^{-3}$ (unpaired *t*-test).

C Relative levels of DNA-binding fluorescence as compared to $YAP^{-/-}$ by dividing the average fluorescence level of each condition in (B) by the average fluorescence of $YAP^{-/-}$.

D Western blots of different MKN28 cell lines. eGFP-YAP and optoYAP lines are transfected into the $YAP^{-/-}$ background.
