# Supplementary figures and images for: Optogenetic control of YAP cellular localisation and function
Source: EMBO Rep. 2022 Jul 25;23(9):e54401. doi: 10.15252/embr.202154401 (PMC9442306; doi:10.15252/embr.202154401)

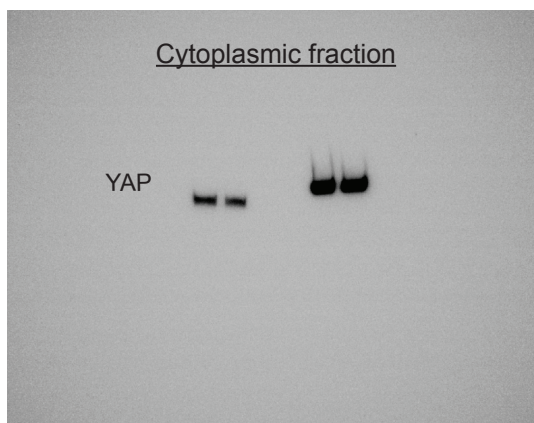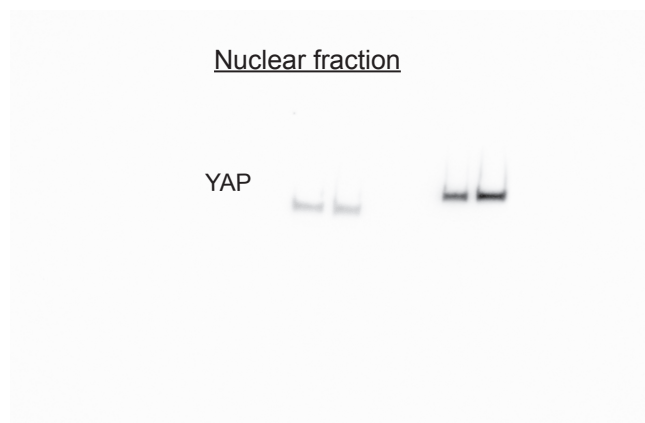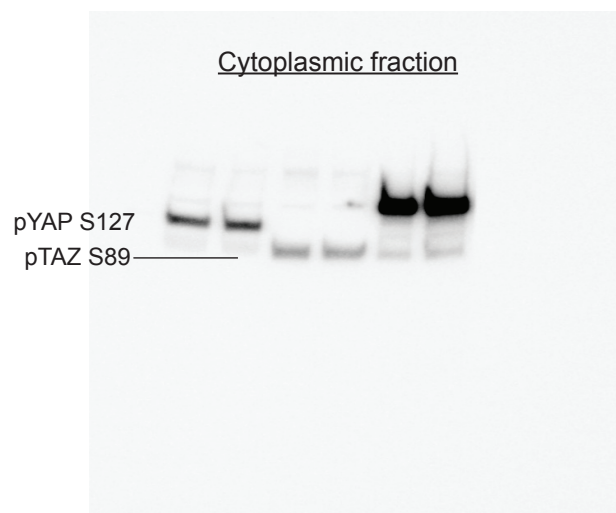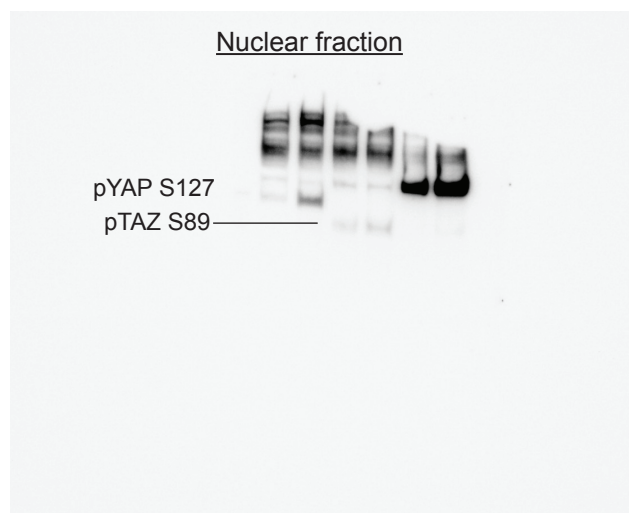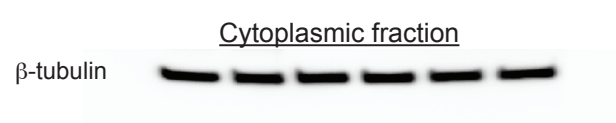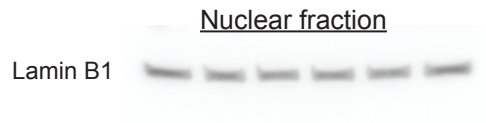

Unedited and uncropped blots from Fig. EV1E

Supplement: Supplementary file 6 — Source Data for Expanded view [file EMBR-23-e54401-s004.zip › EMBR_2204_Fig. EV1E Source Data.pdf]
