# Supplementary material for: Optogenetic control of YAP cellular localisation and function
Source: EMBO Rep. 2022 Jul 25;23(9):e54401. doi: 10.15252/embr.202154401 (PMC9442306; doi:10.15252/embr.202154401)
Supplement: Supplementary file 7 [file EMBR-23-e54401-s005.pdf]

EMBO reports

Optogenetic control of YAP cellular localisation and function

Pearlyn Toh, Jason Lai, Anke Hermann, Olivier Destaing, Michael Sheetz, Marius Sudol, and Timothy Saunders
DOI: 10.15252/embr.202154401

Corresponding author(s): Timothy Saunders (timothy.saunders@warwick.ac.uk) , Michael Sheetz (misheetz@utmb.edu), Marius Sudol (mbims@nus.edu.sg)

Review Timeline:

Submission Date:	28th Nov 21
Editorial Decision:	18th Jan 22
Revision Received:	21st Apr 22
Editorial Decision:	10th Jun 22
Revision Received:	17th Jun 22
Accepted:	1st Jul 22

Editor: Martina Rembold

Transaction Report:

(Note: With the exception of the correction of typographical or spelling errors that could be a source of ambiguity, letters and reports are not edited. Depending on transfer agreements, referee reports obtained elsewhere may or may not be included in this compilation. Referee reports are anonymous unless the Referee chooses to sign their reports.)

1st Editorial Decision **18th Jan 2022**

Dear Timothy,

Thank you for the submission of your research manuscript to our journal. We have now received the full set of referee reports that is copied below.

As you will see, the referee opinions on your study are divided.
Clearly, both referee 1 and 2 notice a considerable amount of background activation of optoYAP in the dark. Referee 1 considers it essential to provide proof that optoYAP is not toxic and does not interfere with normal zebrafish development by establishing a transgenic line. Certainly, such a line would be the best possible option and would also present a very useful tool for the community. However, after discussing the issue further with my colleagues and after receiving further feedback from referee 2, we suggest addressing the background effects further using transient expression. Referee 2 suggested: "[...] perhaps an alternative to making a transgenic line would be to include rigorous description of the background effects and demonstrate how these can be controlled during experiments."

On balance, we have decided to invite you to revise your study for potential publication in EMBO Reports. It will be important to address background activation of optoYAP in the dark and to provide further validation and characterization of the tool, along the lines suggested by the referees. RNA-FISH analysis of YAP target gene expression and data on whether the tool can be used in e.g. a delineated area of zebrafish tissue could also be provided using transient expression approaches, given that the proper controls are provided.

Taken together, we would like to invite you to revise your manuscript with the understanding that the referee concerns (as detailed above and in their reports) must be fully addressed and their suggestions taken on board. Please address all referee concerns in a complete point-by-point response. Acceptance of the manuscript will depend on a positive outcome of a second round of review. It is EMBO reports policy to allow a single round of revision only and acceptance or rejection of the manuscript will therefore depend on the completeness of your responses included in the next, final version of the manuscript.

We realize that it is difficult to revise to a specific deadline. In the interest of protecting the conceptual advance provided by the work, we recommend a revision within 3 months (April 18th, 2022). Please discuss the revision progress ahead of this time with the editor if you require more time to complete the revisions.

You can either publish the study as a short report or as a full article. For short reports, the revised manuscript should not exceed 27,000 characters (including spaces but excluding materials & methods and references) and 5 main plus 5 expanded view figures. The results and discussion sections must further be combined, which will help to shorten the manuscript text by eliminating some redundancy that is inevitable when discussing the same experiments twice. For a normal article there are no length limitations, but it should have more than 5 main figures and the results and discussion sections must be separate. In both cases, the entire materials and methods must be included in the main manuscript file.

IMPORTANT NOTE:
We perform an initial quality control of all revised manuscripts before re-review. Your manuscript will FAIL this control and the handling will be DELAYED if the following APPLIES:

1) A data availability section providing access to data deposited in public databases is missing. If you have not deposited any data, please add a sentence to the data availability section that explains that.

2) Your manuscript contains statistics and error bars based on n=2. Please use scatter blots in these cases. No statistics should be calculated if n=2.

When submitting your revised manuscript, please carefully review the instructions that follow below. Failure to include requested items will delay the evaluation of your revision.

When submitting your revised manuscript, we will require:

1) a .docx formatted version of the manuscript text (including legends for main figures, EV figures and tables). Please make sure that the changes are highlighted to be clearly visible.

2) individual production quality figure files as .eps, .tif, .jpg (one file per figure).
Please download our Figure Preparation Guidelines (figure preparation pdf) from our Author Guidelines pages https://www.embopress.org/page/journal/14693178/authorguide for more info on how to prepare your figures.

3) a .docx formatted letter INCLUDING the reviewers' reports and your detailed point-by-point responses to their comments. As part of the EMBO Press transparent editorial process, the point-by-point response is part of the Review Process File (RPF),

which will be published alongside your paper.

4) a complete author checklist, which you can download from our author guidelines (). Please insert information in the checklist that is also reflected in the manuscript. The completed author checklist will also be part of the RPF.

5) Please note that all corresponding authors are required to supply an ORCID ID for their name upon submission of a revised manuscript (). Please find instructions on how to link your ORCID ID to your account in our manuscript tracking system in our Author guidelines
()

6) We replaced Supplementary Information with Expanded View (EV) Figures and Tables that are collapsible/expandable online. A maximum of 5 EV Figures can be typeset. EV Figures should be cited as 'Figure EV1, Figure EV2" etc... in the text and their respective legends should be included in the main text after the legends of regular figures.

- For the figures that you do NOT wish to display as Expanded View figures, they should be bundled together with their legends in a single PDF file called *Appendix*, which should start with a short Table of Content. Appendix figures should be referred to in the main text as: "Appendix Figure S1, Appendix Figure S2" etc. See detailed instructions regarding expanded view here:

- Additional Tables/Datasets should be labeled and referred to as Table EV1, Dataset EV1, etc. Legends have to be provided in a separate tab in case of .xls files. Alternatively, the legend can be supplied as a separate text file (README) and zipped together with the Table/Dataset file.

7) Please note that a Data Availability section at the end of Materials and Methods is now mandatory. In case you have no data that requires deposition in a public database, please state so instead of refereeing to the database.
See also < https://www.embopress.org/page/journal/14693178/authorguide#dataavailability>). Please note that the Data Availability Section is restricted to new primary data that are part of this study.

8) Figure legends and data quantification:
The following points must be specified in each figure legend:
- the name of the statistical test used to generate error bars and P values,
- the number (n) of independent experiments (please specify technical or biological replicates) underlying each data point,
- the nature of the bars and error bars (s.d., s.e.m.)
- If the data are obtained from n {less than or equal to} 2, use scatter blots showing the individual data points.
Discussion of statistical methodology can be reported in the materials and methods section, but figure legends should contain a basic description of n, P and the test applied.
See also the guidelines for figure legend preparation:
https://www.embopress.org/page/journal/14693178/authorguide#figureformat
- Please also include scale bars in all microscopy images.

9) We would also encourage you to include the source data for figure panels that show essential data. Numerical data should be provided as individual .xls or .csv files (including a tab describing the data). For blots or microscopy, uncropped images should be submitted (using a zip archive if multiple images need to be supplied for one panel). Additional information on source data and instruction on how to label the files are available .

10) Our journal encourages inclusion of *data citations in the reference list* to directly cite datasets that were re-used and obtained from public databases. Data citations in the article text are distinct from normal bibliographical citations and should directly link to the database records from which the data can be accessed. In the main text, data citations are formatted as follows: "Data ref: Smith et al, 2001" or "Data ref: NCBI Sequence Read Archive PRJNA342805, 2017". In the Reference list, data citations must be labeled with "[DATASET]". A data reference must provide the database name, accession number/identifiers and a resolvable link to the landing page from which the data can be accessed at the end of the reference. Further instructions are available at .

11) As part of the EMBO publication's Transparent Editorial Process, EMBO reports publishes online a Review Process File to accompany accepted manuscripts. This File will be published in conjunction with your paper and will include the referee reports, your point-by-point response and all pertinent correspondence relating to the manuscript.

You are able to opt out of this by letting the editorial office know (emboreports@embo.org). If you do opt out, the Review Process File link will point to the following statement: "No Review Process File is available with this article, as the authors have chosen not to make the review process public in this case."

We would also welcome the submission of cover suggestions, or motifs to be used by our Graphics Illustrator in designing a cover.

I look forward to seeing a revised form of your manuscript when it is ready and please let me know if you have questions or comments regarding the revision.

Kind regards,

Martina

Martina Rembold, PhD
Senior Editor
EMBO reports

****

Referee #1:

Toh and co-workers report an optoYAP construct that allows light-dependent manipulation of YAP nuclear import. They employ the LINuS optogenetic module, previously developed by the DiVentura lab, which consists in the photo-uncaging of a nuclear localization signal using blue light. Transient transfection in cell culture and mRNA injection in zebrafish embryos demonstrate an increase in target gene expression after 48h of light induction. From the images presented, optoYAP appears to be localized to the cytoplasm but also in the nucleus in the dark (Fig1B and 3B). Measuring downstream target gene expression after 48h of transfection by pooling many cells together will average out the dark state activity of the construct (i.e. the extent to which YAP target genes are expressed in the dark as a consequence of the ineffective nuclear exclusion of optoYAP). The experiments performed in fish embryos using optoYAP mRNA injection (Fig3), demonstrate also nuclear localization of optoYAP in the dark, and the expression of target genes after 10h of illumination does not allow assessing the efficacy and specificity of this tool for the same reasons mentioned above. What is needed to demonstrate that the dark state of optoYAP does not interfere with normal animal physiology is the generation of an optoYAP transgenic line at the very minimum. This will allow a more precise analysis of YAP target expressions at the single cell level (e.g. quantitative RNA-FISH or live imaging) and on shorter time scales (minutes not hours). In summary, at this stage this study provides limited new information both from the technological or biological standpoint, and it is not clear this tool will allow us to learn novel insight into YAP function. I think part of the problem relates to the use of the LINuS system which does not allow efficient trapping of protein in the cytoplasm (see also work from El-Samad lab, e.g. Chen YS Cell Systems 2021). Along this line, a recent publication by Dowbay et al JCS 2021 demonstrates a more elaborated strategy to effectively control YAP nuclear-cytoplasmic shuttling with light.

Referee #2:

The manuscript describes a LOV2 based blue-light sensitive optogenetic system for artificially activating YAP signalling: Under blue light the LOV2 domain unfolds, exposing the caged NLS and transporting a full-length fusion protein for YAP to the nucleus, where it initiates signalling. The authors have demonstrated functionality in cell culture and in zebrafish embryos. They show the upregulation of downstream target genes and the regulation of cell colony growth in response to optogenetic activation of YAP. The functionality of this optogenetic tool will be of interest to the many researchers studying Hippo and YAP signalling. The evidence for functionality of the tool is compelling. I have several minor comments that the authors should address to ensure that a full understanding of the tool's functionality and potential applications is conveyed. E.g. how variable the levels of activation are between cells, how deep within zebrafish embryos the tool is effective, what is the level of background activation generated by overexpression of full-length YAP fusion-protein.

Presentation and analysis comments:
• It is not clear for non-experts what the consequences are of the pathways described in the first paragraph. I suggest the key information to make clear here is that nuclear localisation of YAP is related to its activity (e.g. like the sentence in line 124).

• Please specify the macro used in line 441. Also there is a 'source code legend' but maybe I missed where the source code is in the files - I couldn't see it.

• Please specify the statistical analysis used in each figure legend or in the methods. It is not currently possible to review whether the correct statistical tests were applied (e.g. if variances are different between groups). For curve fitting, it would be helpful for non-specialists to define each term in the equations. Please define 'biological replicates'.

• It would be helpful to label axes with the specific parameter being measured. E.g. 'DNA-binding fluorescence' rather than just 'fluorescence'.

Functionality comments:
• The images shown in figure 1D and S1A appear to illustrate a higher overall level MCherry following each cycle of activation/recovery compared with levels before activation. Can the authors clarify and, if appropriate, quantify what is happening

there?

• Line 97: "the light intensities used are substantially lower than those reported to induce phototoxicity". What were the intensities used? It would be clearer to include them in the text here.

• Figure 2A-C suggests that there is not much background activation from the optoYAP construct. This is good but why does overexpression of YAP fusion protein not cause a large background effect? Is it due to the NES sequence? This would be useful to elaborate on for researchers who may want to construct similar optogenetic constructs. However, there does seem to be a small background effect present (e.g. in total DNA over time figure 2B-C). can the authors check whether this is significant? Since the NES does not seem to work in zebrafish, does this mean that there are relatively higher levels of background activation in zebrafish than in cell culture?

• Line 142. What is hYAP and what is fYAP? Do you mean that the zebrafish-specific sequence was used for the fYAP sequence? If not then what is the difference between the 2 versions of OptoYAP?

• Line 147: 'deep cells'. How deep is this and is there a limit at which nuclear localisation of YAP is not seen? What does the nuclear localisation look like over the z-axis of the embryo at later stages (e.g. the 10-16hpf stages analysed in figure 3)? It is important to know whether this technique is functional in the whole depth of the embryo (and therefore in all tissues) or is limited to relatively superficial cells.

• If optoYAP is expressed transiently then expression levels will vary between cells. As well as N/C ratio or relative fold change, it would therefore be useful to see how the absolute levels of expression vary between cells and with and without optoYAP before/after activation. A discussion of how variation in YAP activation might be eliminated (e.g. by feedback control of light intensity: doi: 10.1038/nmeth.1700) would be helpful.

• MKN28 transformed cell analysis (line 173 onwards): the analysis in figure 4C & D suggests that there is some background activation of optoYAP, since the colony size is higher in optoYAP transfected YAP-/-cells without light than in untransfected YAP-/- cells. I also don't understand why higher proliferation would lead to more colonies. Doesn't each colony arise from a single cell that then proliferates? Did the authors seed the same number of cells each time for the colony analysis?

• The cells were exposed to 1s of blue light every 30s for 1 week in some cases (e.g. figure 2B&C). The read out of these experiments was DNA binding fluorescence, which didn't appear to bleach under this light exposure regime according to the graphs. It would be helpful to know whether the MCh signal from the OptoYAP was still visible and reversible after such a long period of light activation.

• The Dowbaj et al reference in now in press: doi: 10.1242/jcs.253484. It seems that this system might have a different application e.g. 'measuring the rates of nuclear entry and exit' to the one presented in the current manuscript, which is more focused on artificially over-activating YAP signalling. It would be helpful to readers to have a more balanced discussion of when each system might be used.

• The reference 38 seems out of place in line 224: "These features add to the ease of use over other optogenetic approaches that multiple components or activation in the infrared spectrum". Uda et al 2017 specifically describes the synthesis of the chromophore necessary for the Phytochrome optogenetics system. Perhaps a review comparing multiple systems would be better placed here.

• Line 234: "This sequence divergence suggests that the nuclear import/export machineries in the zebrafish may be more sensitive to modified c-Myc NLS and could explain the differing dynamics of optoYAP between human and zebrafish cells." Can the authors explain why the lack of homology between zebrafish and human NLS and NES sequences might lead to a higher, rather than lower efficiency of nuclear import/export?

Referee #3:

The YAP/TAZ family of transcriptional co-activators are key components of the Hippo pathway that regulate cell proliferation and organ growth. This study develops a single-component optogenetic tool that enables YAP nuclear localization and activity upon light stimulation. The authors characterize this new reagent in HEK293T cells and then demonstrates its applicability in Zebrafish embryos and in anchorage independent growth in other cell culture lines. Overall, the study was straightforward and the conclusions were appropriate. I only have one main recommendation and a few minor points.

Main recommendation:
1) The authors conclude that the effects of this optogenetic tool reflect the 'activation' of YAP/TAZ. 'Proof' that this tool toggles 'on' the Hippo pathway and does not result in a broader dysregulation of transcription, would be to show that changes in gene expression are specific to TEAD targets or that changes in gene expression/cell growth depend on TEAD transcription factors. Inclusion of either of these points would be stronger evidence arguing that the optogenetic tool functions in the context of the

native Hippo pathway.

Minor points:
1) Line 52-54: The authors cite numerous examples of optogenetic strategies with spatiotemporal control. Another excellent, recent example that nicely makes the authors point was Cavanaugh et al., Developmental Cell, 2020.
2) Figure 1B and 1C: For these two panels, the configuration of cells and the relative accumulation of nuclear YAP for the 0 min time points. Are the same cells being used? If so, this should be noted in the figure legend for transparency.

We thank the Reviewers for their constructive critiques of our original submission. We have now clarified the important points raised and improved the data analysis, including adding more cells. We hope that with these changes, the manuscript is suitable for acceptance. In the revised manuscript, changes are highlighted in red. Below, we detail in blue our responses to the specific points highlighted by the Reviewers.

Referee #1:

Toh and co-workers report an optoYAP construct that allows light-dependent manipulation of YAP nuclear import. They employ the LINuS optogenetic module, previously developed by the DiVentura lab, which consists in the photo-uncaging of a nuclear localization signal using blue light. Transient transfection in cell culture and mRNA injection in zebrafish embryos demonstrate an increase in target gene expression after 48h of light induction.

We thank the Reviewer for their insights and suggestions. We detail specific replies below.

From the images presented, optoYAP appears to be localized to the cytoplasm but also in the nucleus in the dark (Fig1B and 3B).

For optoYAP in tissue culture cells, the optogenetic construct is excluded from the nucleus when cells are in the dark, as seen in Fig. 1D. We do see baseline variation between cells, likely due to different transfection levels between cells. However, we always see clear changes in the nuclear signal upon activation (Reviewer Fig. 1).

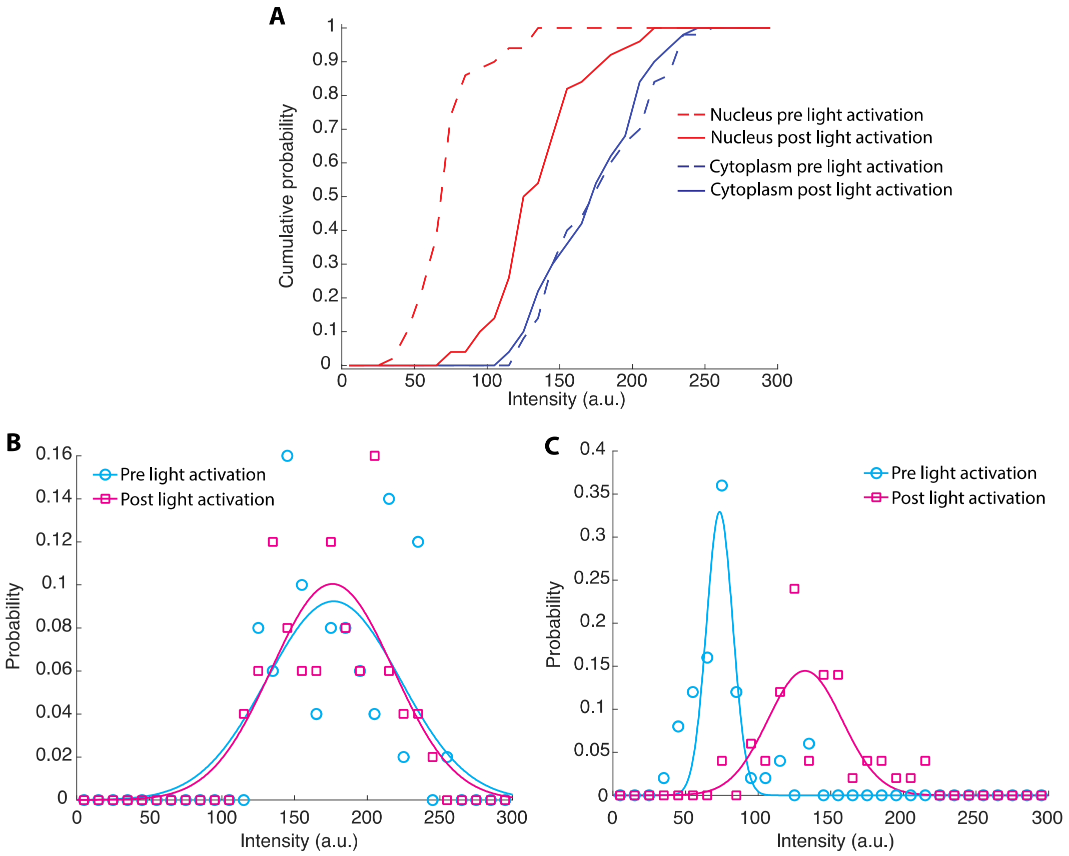

Reviewer Figure 1. (A) Graph showing cumulative distribution of nuclear and cytoplasmic intensity of 50 cells before and after light activation protocol. **(B-C)** Histogram of **(B)** cytoplasmic and **(C)** nuclear intensity of the same 50 cells, binned into 10 a.u. bins.

Measuring downstream target gene expression after 48h of transfection by pooling many cells together will average out the dark state activity of the construct (i.e. the extent to which YAP target genes are expressed in the dark as a consequence of the ineffective nuclear exclusion of optoYAP).
We agree that our pooling averages out cells that may have higher nuclear signal. However, in our qPCR data of downstream target genes in Fig. 2A, there is a clear distinction between the cells kept in dark as compared to cells kept under light activation. Therefore, although we do see some nuclear localised signal - especially in cells with high expression levels of optoYAP – the changes in target gene expression are stark and so low levels of nuclear localisation appears to not alter our conclusions.

The experiments performed in fish embryos using optoYAP mRNA injection (Fig3), demonstrate also nuclear localization of optoYAP in the dark, and the expression of target genes after 10h of illumination does not allow assessing the efficacy and specificity of this tool for the same reasons mentioned above. What is needed to demonstrate that the dark state of optoYAP does not interfere with normal animal physiology is the generation of an optoYAP transgenic line at the very minimum. This will allow a more precise analysis of YAP target expressions at the single cell level (e.g. quantitative RNA-FISH or live imaging) and on shorter time scales (minutes not hours). In summary, at this stage this study provides limited new information both from the technological or biological standpoint, and it is not clear this tool will allow us to learn novel insight into YAP function.
We acknowledge that for optofYap in the zebrafish embryos (Fig. 3B), the recombinant protein is generally homogenously distributed throughout the cells when kept in the dark. However, after 20 mins of pulsed light activation, there is an unmistakable accumulation of the optogenetic construct in the nucleus as compared to the dark state. Therefore, there is a clear change, and this change is also shown in the qPCR results.

Nonetheless, we agree that having a zebrafish line stably expressing optofYap is the 'gold standard' to evaluate our optogenetic construct *in vivo*. Therefore, we have generated a transgenic zebrafish line expressing optofYap in cardiomyocytes by cloning optofYap into a heart specific *myl7* promoter. These fish were created by injecting myl7:optofYap into wildtype zebrafish embryos and screening embryos with positive mCherry signal in cardiomyocytes 3 days post fertilisation. Positive clones selected were raised and outcrossed to wildtype fish after 3 months. Embryos were screen again for mCherry signal in cardiomyocytes and raised to maturity. Using the F2 generation transgenic fish, we outcrossed them to wildtype and imaged hearts of fish kept in the light (Reviewer Fig. 2) or in the dark (Reviewer Fig. 3) at 3 days post fertilisation. We see evidence for light-dependent control of Yap localisation. However, this line requires more detailed characterisation and optimisation, which we believe is beyond the scope of this manuscript and warrants a paper on its own (where we aim to study the role of YAP in heart regeneration). Reviewer Fig. 2 and 3 demonstrate that such an approach is feasible with optofYap.

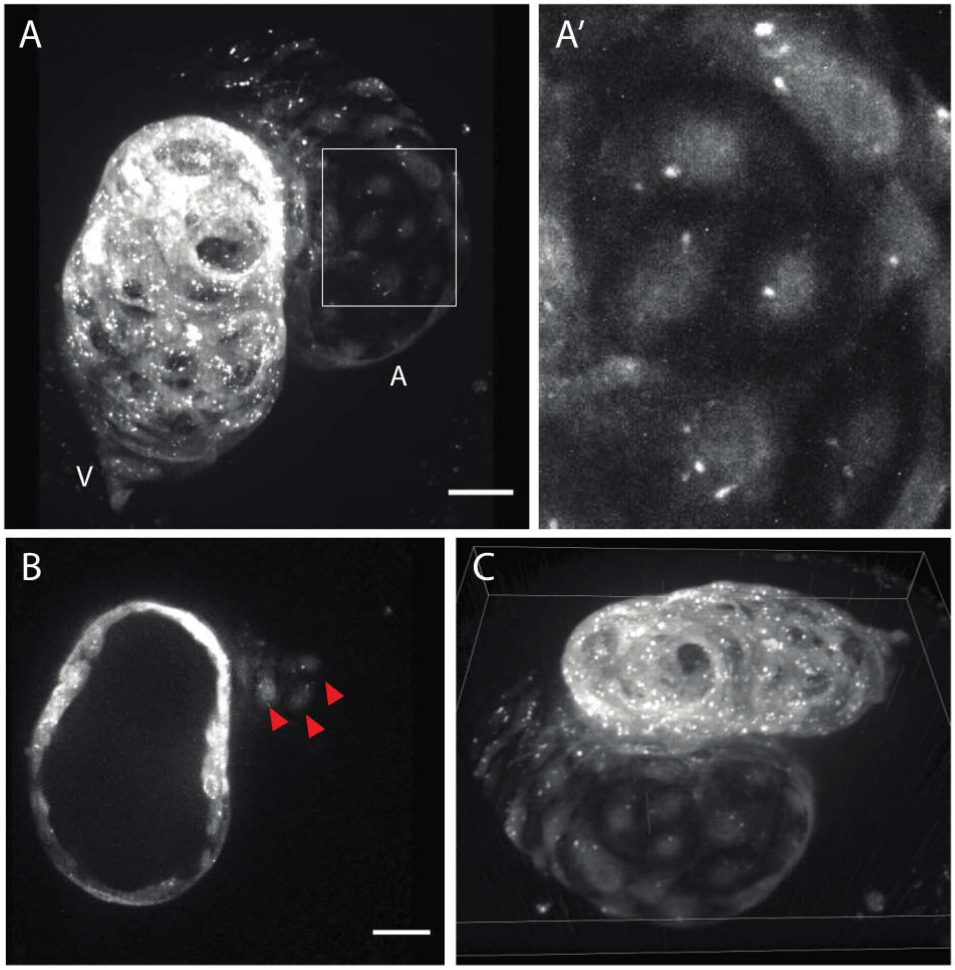

Reviewer Figure 2. Representative images of cardiomyocytes expressing myl7:optofYap in 3 day old zebrafish hearts kept in light. (A) Maximum intensity projection of 100 z-stacks of cardiac tissue. Atrium and ventricle are labelled as 'A' and 'V'. Scale bars, 100 µm. **(A')** Enlarged view of boxed region in (A), showing nuclear localisation of myl7:optofYap in various cells. **(B)** Single z-slice of cardiac tissue with red arrow heads highlighting cells with nuclear translocation of optofYap in the atrium. Scale bars, 100 µm. **(C)** 3D projection of cardiac tissue.

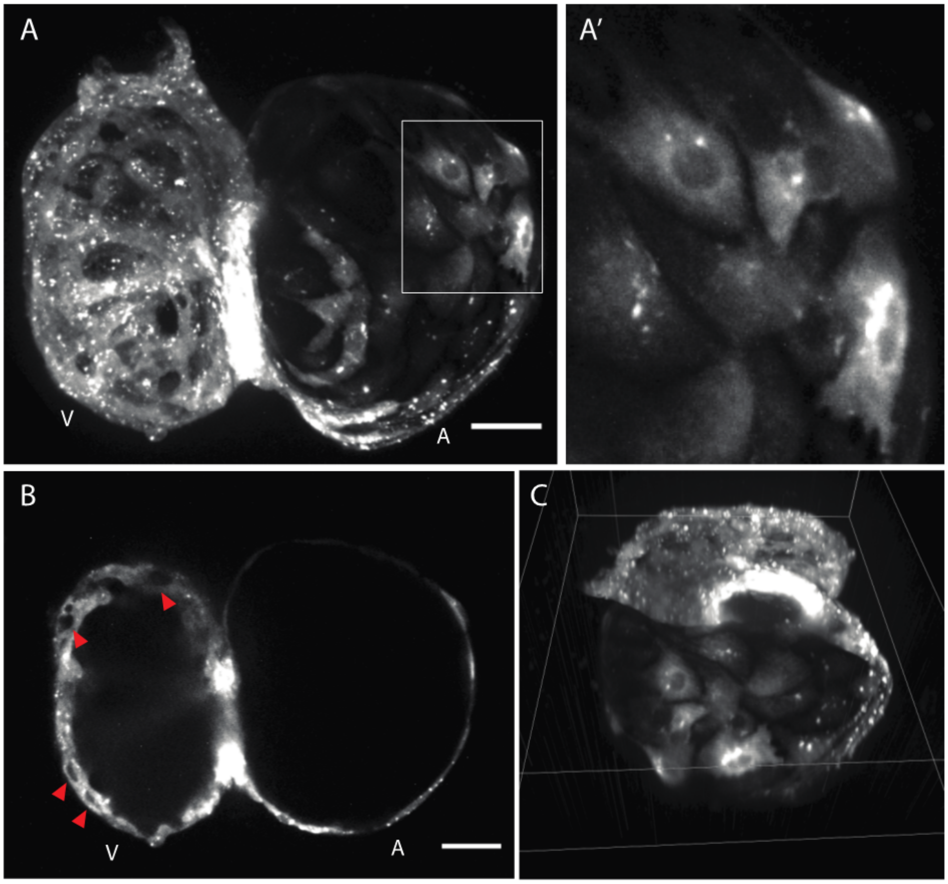

Reviewer Figure 3. Representative images of cardiomyocytes expressing myl7:optofYap in 3 day old zebrafish hearts kept in the dark. (A) Maximum intensity projection of 100 z-stacks of cardiac tissue. Atrium and ventricle are labelled as 'A' and 'V'. Scale bars, 100 µm. **(A')** Enlarged view of boxed region in (A), showing nuclear exclusion of myl7:optofYap. **(B)** Single z-slice of cardiac tissue with red arrow heads highlighting cells with nuclear exclusion of optofYap in the ventricle. Atrium and ventricle are labelled as 'A' and 'V'. Scale bars, 100 µm. **(C)** 3D projection of cardiac tissue.

I think part of the problem relates to the use of the LINuS system which does not allow efficient trapping of protein in the cytoplasm (see also work from El-Samad lab, e.g. Chen YS Cell Systems 2021). Along this line, a recent publication by Dowbay et al JCS 2021 demonstrates a more elaborated strategy to effectively control YAP nuclear-cytoplasmic shuttling with light.

We have included now a more complete discussion of Dowbaj *et al*. that is now in press. We agree that Dowbaj *et al*. describe an elegant optogenetic YAP to control localisation between the mitochondria and nucleus. However, the authors did not demonstrate that this approach was functional – in the sense of activating downstream YAP targets - once it is translocated into the nucleus. Instead, Dowbaj *et al.* tackle a complementary problem; describing the rates of import and export of various YAP mutants. The strength of our paper lies in the functional assays we have shown with nuclear-localised optogenetic YAP, which is an essential characteristic to employ such optogenetic tools to study the role of the Hippo-YAP pathway in growth regulation. Therefore, the two approaches have different advantages and do not conflict with each other.

Referee #2:

The manuscript describes a LOV2 based blue-light sensitive optogenetic system for artificially activating YAP signalling: Under blue light the LOV2 domain unfolds, exposing the caged NLS and transporting a full-length fusion protein for YAP to the nucleus, where it initiates signalling. The authors have demonstrated functionality in cell culture and in zebrafish embryos. They show the upregulation of downstream target genes and the regulation of cell colony growth in response to optogenetic activation of YAP. The functionality of this optogenetic tool will be of interest to the many researchers studying Hippo and YAP signalling. The evidence for functionality of the tool is compelling.

We thank the Reviewer for the overall positive view on our manuscript.

I have several minor comments that the authors should address to ensure that a full understanding of the tool's functionality and potential applications is conveyed. E.g. how variable the levels of activation are between cells, how deep within zebrafish embryos the tool is effective, what is the level of background activation generated by overexpression of full-length YAP fusion-protein.

We outline our responses to these points below.

Presentation and analysis comments:

• It is not clear for non-experts what the consequences are of the pathways described in the first paragraph. I suggest the key information to make clear here is that nuclear localisation of YAP is related to its activity (e.g. like the sentence in line 124).

We have added a sentence to elaborate on the effect of nuclear localised YAP (Line 41-42).

• Please specify the macro used in line 441. Also there is a 'source code legend' but maybe I missed where the source code is in the files - I couldn't see it.

The macro used is a plugin for Fiji and was written by a collaborator, Dr Richard De Mets, as noted in the 'Acknowledgements' section. We clarified that the macro is written by a collaborator (Line 418-419). The macro calculates the nuclear to cytoplasmic intensity using the mean intensity of each region. Each nuclear and cytoplasmic region of every time frame and cell are hand drawn.

The source code is the code of the macro that we have originally submitted to EMBO Reports, and we are not aware if it has been shared with the reviewers or not.

• Please specify the statistical analysis used in each figure legend or in the methods. It is not currently possible to review whether the correct statistical tests were applied (e.g. if variances are different between groups). For curve fitting, it would be helpful for non-specialists to define each term in the equations. Please define 'biological replicates'.

The different statistical tests used have been added to each figure legends where applicable. 'Biological replicates' has been defined in the methods (Line 531-533) for clarity.

For curve fitting, we take a simple exponential model for activation. The formulae for fitting are provided in the appropriate supplementary (expanded view) figures. In these equations, typically a represents a basal level of signal intensity, b is the multiplicative factor representing the change in signal due to light activation, and τ represents the time scale over which the signal changes before/after light activation. These terms have now been clarified in the legends for Figures EV1 and

EV2. Fitting was performed using the *fit* function in Matlab, with the corresponding confidence intervals given in brackets in the supplementary (expanded view) figures.

• It would be helpful to label axes with the specific parameter being measured. E.g. 'DNA-binding fluorescence' rather than just 'fluorescence'.
The axes in Figure 2B and 2C, and Figure EV3A and EV3B have been corrected.

Functionality comments:
• The images shown in figure 1D and S1A appear to illustrate a higher overall level MCherry following each cycle of activation/recovery compared with levels before activation. Can the authors clarify and, if appropriate, quantify what is happening there?
We have checked the images and the contrasts of each frame was different, which resulted in the ambiguity of mCherry levels. We have corrected Figure 1D and Figure EV1A and we thank the Reviewer for their careful observation.

• Line 97: "the light intensities used are substantially lower than those reported to induce phototoxicity". What were the intensities used? It would be clearer to include them in the text here.
We have included the light intensity used in the sentence to clarify the text (Line 99). We have also made clear that such intensities are unlikely to have a phenotypic effect in their own right.

• Figure 2A-C suggests that there is not much background activation from the optoYAP construct. This is good but why does overexpression of YAP fusion protein not cause a large background effect? Is it due to the NES sequence? This would be useful to elaborate on for researchers who may want to construct similar optogenetic constructs. However, there does seem to be a small background effect present (e.g. in total DNA over time figure 2B-C). can the authors check whether this is significant?
The optogenetic backbone contains a NES that is not photocaged in the dark, allowing endogenous exportins to bind to the sequence and transport optoYAP out of the nucleus. Of course, as highlighted by Reviewer 1, this effect is not perfect and there are occasional cells with clear optoYAP signal in the nucleus, even without light activation. However, for example, the background effect present in Fig. 2B and 2C of cells transfected with optoYAP kept in the dark, is not significant as compared to cells that were untransfected. The results of the statistical test have been added to the figure for clarity.

Since the NES does not seem to work in zebrafish, does this mean that there are relatively higher levels of background activation in zebrafish than in cell culture?
It is correct that from our imaging (e.g. Fig. 3B) optofYap is more homogenous throughout each cell despite being kept in the dark when compared to cell culture.

Comparing the downstream gene expression levels of optofYap in zebrafish (Fig. 3D) and optoYAP in cultured cells (Fig. 2A), there is a higher gene expression level in zebrafish embryos kept in the dark (Fig. 3D) in contrast to cells kept in the dark (Fig. 2A). Therefore, there appears to be a slightly higher level of background activation in the zebrafish embryos than in cell culture (as evidenced in the qPCR data). Importantly, light activation shows a clear change in downstream target gene expression.

• Line 142. What is hYAP and what is fYAP? Do you mean that the zebrafish-specific sequence was used for the fYAP sequence? If not then what is the difference between the 2 versions of OptoYAP?
hYAP refers to human YAP and fYap refers to fish Yap (specific to *Danio rerio*), which have slightly different sequences but are largely conserved (Hu *et al.*, 2013), with identical key elements such as TEAD-binding domain and WW domains. In the zebrafish experiments, we modified the optogenetic construct to replace hYAP with fYap, with all other domains of the optogenetic backbone unchanged.

• Line 147: 'deep cells'. How deep is this and is there a limit at which nuclear localisation of YAP is not seen? What does the nuclear localisation look like over the z-axis of the embryo at later stages (e.g. the 10-16hpf stages analysed in figure 3)? It is important to know whether this technique is functional in the whole depth of the embryo (and therefore in all tissues) or is limited to relatively superficial cells.
The 'deep cells' are named because they are situated below the enveloping layer (EVL), which is only one cell thick (2 microns). We imaged the embryos up to 10 microns deep. The specificity of the optogenetic activation is limited by dispersal of the laser beam as we go deeper into the embryo. However, we highlight the data in Reviewer Fig. 2 and 3, where we show that this technique can be functional in the entire organ of the heart, which lies deeper within the embryo.

• If optoYAP is expressed transiently then expression levels will vary between cells. As well as N/C ratio or relative fold change, it would therefore be useful to see how the absolute levels of expression vary between cells and with and without optoYAP before/after activation.
We agree with the Reviewer and have gone back and reanalysed the data. From the nucleus and cytoplasmic intensity data used to generate the N/C ratio and relative fold change, we plotted the absolute values of all 50 cells analysed before and after light activation (Reviewer Fig. 1).

A discussion of how variation in YAP activation might be eliminated (e.g. by feedback control of light intensity: doi: 10.1038/nmeth.1700) would be helpful.
We agree that this approach is important for reducing error in long time activation of embryos and cell culture. For the experiments outlined here, we look at relatively short periods of activation. However, for our future work on the role of YAP in the heart, such approaches are worth considering. We have included a brief discussion on this point in the revised manuscript (Lines 286-288).

• MKN28 transformed cell analysis (line 173 onwards): the analysis in figure 4C & D suggests that there is some background activation of optoYAP, since the colony size is higher in optoYAP transfected YAP-/-cells without light than in untransfected YAP-/- cells. I also don't understand why higher proliferation would lead to more colonies. Doesn't each colony arise from a single cell that then proliferates? Did the authors seed the same number of cells each time for the colony analysis?
Activated optoYAP is nuclear localised thus increasing downstream gene expression required for proliferation (CTGF and CYR61, as seen in Fig. 2A). Boosting the expression of proliferative genes in these cells increases colony formation in soft agar.

Cells were grown to 80% confluency in a T25 flask prior to the experiment. Cells were trypsinised and suspended, and counted manually in a hemocytometer to determine the concentration of cells. The same number of cells were seeded in each well for the colony formation assay and we have

added a sentence in Methods for clarification (Line 501-503). The initial cell numbers being seeded for every condition were very similar and repeatable. Therefore, the enhanced colony formation seen under light activation of optoYAP can be linked to the increased transcription of proliferative genes in these cells.

• The cells were exposed to 1s of blue light every 30s for 1 week in some cases (e.g. figure 2B&C). The read out of these experiments was DNA binding fluorescence, which didn't appear to bleach under this light exposure regime according to the graphs. It would be helpful to know whether the MCh signal from the OptoYAP was still visible and reversible after such a long period of light activation.

We checked the viability of cells after each long-term light exposure, in this case 1 week. The cells looked healthy, and we did not notice significant apoptosis nor phenotypic variation. After one week of pulsed light exposure, the mCherry signal is still visible and reversible, though unfortunately we do not keep this data.

• The Dowbaj et al reference in now in press: doi: 10.1242/jcs.253484. It seems that this system might have a different application e.g. 'measuring the rates of nuclear entry and exit' to the one presented in the current manuscript, which is more focused on artificially over-activating YAP signalling. It would be helpful to readers to have a more balanced discussion of when each system might be used.

We have edited the reference to reflect the paper in press by Dowbaj et al. The paper by Dowbaj et al. describes an optogenetic YAP and TAZ that is shuttled between the mitochondria and the nucleus. However, a key distinction in our paper is that the optogenetic construct that we have generated is functional when nuclear localised, which the paper by Dowbaj et al. does not show. We believe that tracking the rate of import and export of YAP (which we have also presented) is complementary to demonstrating a functional tool, which we show in our paper in various forms such as downstream gene expression *in vitro* and *in vivo* as well as proliferation and growth assays. As suggested, we have added a paragraph in the manuscript (Line 230-237) to elaborate on the differences between the two systems.

• The reference 38 seems out of place in line 224: "These features add to the ease of use over other optogenetic approaches that multiple components or activation in the infrared spectrum". Uda et al 2017 specifically describes the synthesis of the chromophore necessary for the Phytochrome optogenetics system. Perhaps a review comparing multiple systems would be better placed here.

Thank you for highlighting the misplaced reference. We have updated reference 21 in line 54 and reference 44 in line 227 to better reflect the statements we made.

• Line 234: "This sequence divergence suggests that the nuclear import/export machineries in the zebrafish may be more sensitive to modified c-Myc NLS and could explain the differing dynamics of optoYAP between human and zebrafish cells." Can the authors explain why the lack of homology between zebrafish and human NLS and NES sequences might lead to a higher, rather than lower efficiency of nuclear import/export?

The NLS used in the original paper described by Niopek *et al*. was derived from *Avena sativa*, an oat plant, modifying and adding some amino acid sequences. We picked this particular combination of NLS and NES as it was reported to have the highest nuclear localisation after 488 nm activation. The

basal rate of nuclear export can be interpreted to be lower in zebrafish embryos as compared to tissue culture cells, as seen in the distribution of optogenetic YAP in the dark state (Fig. 1B and Fig. 3B). We have edited this paragraph (Line 247-252) to limit speculation of the strength of NLS and NES in human and zebrafish cells as we did not thoroughly study this aspect of the optogenetic tool generated.

Referee #3:

The YAP/TAZ family of transcriptional co-activators are key components of the Hippo pathway that regulate cell proliferation and organ growth. This study develops a single-component optogenetic tool that enables YAP nuclear localization and activity upon light stimulation. The authors characterize this new reagent in HEK293T cells and then demonstrates its applicability in Zebrafish embryos and in anchorage independent growth in other cell culture lines. Overall, the study was straightforward and the conclusions were appropriate. I only have one main recommendation and a few minor points.

We thank the Reviewer for the generally positive review of our manuscript.

Main recommendation:

1) The authors conclude that the effects of this optogenetic tool reflect the 'activation' of YAP/TAZ. 'Proof' that this tool toggles 'on' the Hippo pathway and does not result in a broader dysregulation of transcription, would be to show that changes in gene expression are specific to TEAD targets or that changes in gene expression/cell growth depend on TEAD transcription factors. Inclusion of either of these points would be stronger evidence arguing that the optogenetic tool functions in the context of the native Hippo pathway.

The three genes assayed in Fig. 2A, *ANKRD1* (Stein *et al.*, 2015), *CTGF* (Zhao *et al.*, 2008) and *CYR61* (Zhang, Pasolli and Fuchs, 2011) are TEAD-mediated YAP transcription targets. We have added this point in the manuscript (Line 125, 128) to clearly highlight that the changes in gene expression are specific to TEAD transcription factors.

To further test the specificity of our conclusions, we have performed another cycle of qPCR looking at 2 genes – *MYC*, another classic YAP-TEAD mediated downstream target (Zanconato *et al.*, 2015), and *EPO*, a growth factor erythropoietin that is not induced by the Hippo-YAP pathway (Singh *et al.*, 2016). It is evident that the optoYAP tool is native to the Hippo-YAP pathway and does not appear to upregulate other growth factors that are not TEAD-mediated (Reviewer Fig. 4). We also include this data in Fig. EV2.

Minor points:

1) Line 52-54: The authors cite numerous examples of optogenetic strategies with spatiotemporal control. Another excellent, recent example that nicely makes the authors point was Cavanaugh et al., Developmental Cell, 2020.

We thank the Reviewer for highlighting this paper to us and we have added a reference to this paper (Line 56).

2) Figure 1B and 1C: For these two panels, the configuration of cells and the relative accumulation of nuclear YAP for the 0 min time points. Are the same cells being used? If so, this should be noted in the figure legend for transparency.

Yes, the same cells are being used. It is noted in the figure legend that the same cells are being used for both panels in Figure 1B and 1C (Line 318).

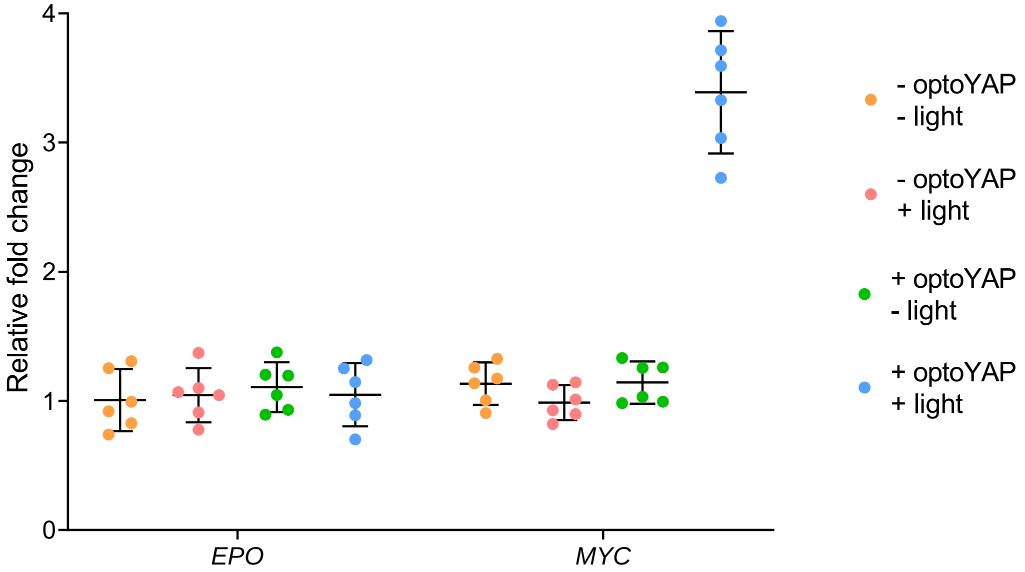

Reviewer Figure 4. qPCR analysis of *EPO* and *MYC*. Erythropoietin (EPO) is a growth factor that is not induced by the Hippo-YAP pathway and does not have an increase in transcript levels with activation of optoYAP. *MYC* is a target gene of YAP-TEAD signalling and is increased in the presence of activated optoYAP.

Bibliography

Hu, J. *et al.* (2013) 'Yes-associated protein (yap) is required for early embryonic development in zebrafish (danio rerio)', *International journal of biological sciences*. 2013/03/06. Ivyspring International Publisher, 9(3), pp. 267–278. doi: 10.7150/ijbs.4887.

Singh, A. *et al.* (2016) 'Hippo Signaling Mediators Yap and Taz Are Required in the Epicardium for Coronary Vasculature Development', *Cell Reports*. Elsevier, 15(7), pp. 1384–1393. doi: 10.1016/j.celrep.2016.04.027.

Stein, C. *et al.* (2015) 'YAP1 Exerts Its Transcriptional Control via TEAD-Mediated Activation of Enhancers', *PLOS Genetics*. Public Library of Science, 11(8), pp. 1–28. doi: 10.1371/journal.pgen.1005465.

Zanconato, F. *et al.* (2015) 'Genome-wide association between YAP/TAZ/TEAD and AP-1 at enhancers drives oncogenic growth', *Nature cell biology*. 2015/08/10, 17(9), pp. 1218–1227. doi: 10.1038/ncb3216.

Zhang, H., Pasolli, H. A. and Fuchs, E. (2011) 'Yes-associated protein (YAP) transcriptional coactivator functions in balancing growth and differentiation in skin', *Proceedings of the National Academy of Sciences*, 108(6), pp. 2270–2275. doi: 10.1073/pnas.1019603108.

Zhao, B. *et al.* (2008) 'TEAD mediates YAP-dependent gene induction and growth control',

Genes and Development, 22(14), pp. 1962–1971. doi: 10.1101/gad.1664408.

Dear Dr. Saunders

Thank you for the submission of your revised manuscript to EMBO reports. We have meanwhile received the report from the referee who was asked to evaluate it.

As you will see, the referee supports publication of the manuscript but recommends extending the discussion and including more information and discussion on basal activation, embryo physiology and spatiotemporal functionality in the manuscript itself, not only in the point-by-point response. Please address this comment and please also include a discussion of the recent study by Iles et al, 2021.

In addition to these points, there are also a few things from the editorial side, that we need before we can proceed with the official acceptance of your study.

- Your manuscript will be published as Report. For short reports, the revised manuscript should not exceed 27,000 characters (including spaces but excluding materials & methods and references) and 5 main plus 5 expanded view figures. The results and discussion sections must further be combined.

- Please note that a Data Availability section at the end of Materials and Methods is now mandatory. In case you have no data that requires deposition in a public database, please state so instead of refereeing to the database.
See also < https://www.embopress.org/page/journal/14693178/authorguide#dataavailability>). Please note that the Data Availability Section is restricted to new primary data that are part of this study.

- Please update the 'Conflict of interest' paragraph to our new 'Disclosure and competing interests statement'. For more information see
https://www.embopress.org/page/journal/14693178/authorguide#conflictsofinterest

- Please update the references to the alphabetical Harvard style. The abbreviation 'et al' should be used if more than 10 authors. You can download the respective EndNote file from our Guide to Authors
https://endnote.com/style_download/embo-reports/

- Movies: Please provide the legend as simple README.txt file and zip this with the movie. The zip files are then uploaded. Please also correct the name to 'Movie EV#'.

- Please remove the Movie and EV Table legends from the manuscript file.

- Source code legend and macro: Please provide reader access to the macro. Either include the entire macro in the methods or upload it as a text file (EV file type).

- The manuscript sections are in the wrong order. Please have a look at our Guide to Authors for more information.
https://www.embopress.org/page/journal/14693178/authorguide#researcharticleguide

- I attach to this email a related manuscript file with comments by our data editors, mainly in the figure legends. Please address all comments and upload a revised file with tracked changes with your final manuscript submission.

- Finally, EMBO reports papers are accompanied online by A) a short (1-2 sentences) summary of the findings and their significance, B) 2-3 bullet points highlighting key results and C) a synopsis image that is 550x200-600 pixels large (width x height) in .png format. You can either show a model or key data in the synopsis image. Please note that the size is rather small and that text needs to be readable at the final size. Please send us this information along with the revised manuscript.

We look forward to seeing a final version of your manuscript as soon as possible.

Kind regards,

Martina Rembold

Martina Rembold, PhD
Senior Editor
EMBO reports

****

Referee #2:

In my view the manuscript is interesting and would be useful to the field, although I think that a fuller explanation within the manuscript in response to the referees' comments (e.g. discussion of basal activation and how this affects the physiology of cells and embryos, demonstration of spatiotemporal functionality), would have improved the impact and novelty of the study. I have also since noticed another manuscript: Illes et al., 2021 PMID: 33718837. This seems more similar to the current tool's functionality than the Dowbaj publication (it activates 'optoYAP' by triggering nuclear translocation and shows downstream functional changes, both in gene expression and in cell growth). This uses a different photoactivation mechanism to the current study.

Dear Martina

Thanks for the positive news. We have made the below requested changes (some comments added below) and I have uploaded onto the website.

We hope the manuscript is now fit for acceptance.

Warm regards

Tim

Dear Dr. Saunders

Thank you for the submission of your revised manuscript to EMBO reports. We have meanwhile received the report from the referee who was asked to evaluate it.

As you will see, the referee supports publication of the manuscript but recommends extending the discussion and including more information and discussion on basal activation, embryo physiology and spatiotemporal functionality in the manuscript itself, not only in the point-by-point response. Please address this comment and please also include a discussion of the recent study by Iles et al, 2021.
We have addressed these concerns. We have added additional material to Figure EV1 and extended the Discussion. We have also clarified a few points regarding the background signal and the dynamics in the main text.

In addition to these points, there are also a few things from the editorial side, that we need before we can proceed with the official acceptance of your study.

- Your manuscript will be published as Report. For short reports, the revised manuscript should not exceed 27,000 characters (including spaces but excluding materials & methods and references) and 5 main plus 5 expanded view figures. The results and discussion sections must further be combined.
The revised version is 23,138 characters, including spaces.

- Please note that a Data Availability section at the end of Materials and Methods is now mandatory. In case you have no data that requires deposition in a public database, please state so instead of refereeing to the database.
See also <
https://www.embopress.org/page/journal/14693178/authorguide#dataavailability><https:

//ddec1-0-en-ctp.trendmicro.com:443/wis/clicktime/v1/query?url=https%3a%2f%2fwww.embopress.org%2fpage%2fjournal%2f14693178%2fauthorguide%23dataavailability%26gt&umid=f0c54d2a-344a-47cf-ac15-5e71402878ad&auth=8d3ccd473d52f326e51c0f75cb32c9541898e5d5-1f96d20ad418c767f1f300011b62e8d8cd8cc11e>;). Please note that the Data Availability Section is restricted to new primary data that are part of this study.

We have now added this statement

- Please update the 'Conflict of interest' paragraph to our new 'Disclosure and competing interests statement'. For more information see https://www.embopress.org/page/journal/14693178/authorguide#conflictsofinterest<https://ddec1-0-en-ctp.trendmicro.com:443/wis/clicktime/v1/query?url=https%3a%2f%2fwww.embopress.org%2fpage%2fjournal%2f14693178%2fauthorguide%23conflictsofinterest&umid=f0c54d2a-344a-47cf-ac15-5e71402878ad&auth=8d3ccd473d52f326e51c0f75cb32c9541898e5d5-6a0275fc602f37e8ed1e0d277e696b49330ee03a>

We have now added this statement

- Please update the references to the alphabetical Harvard style. The abbreviation 'et al' should be used if more than 10 authors. You can download the respective EndNote file from our Guide to Authors
https://endnote.com/style_download/embo-reports/

We have changed the reference style

- Movies: Please provide the legend as simple README.txt file and zip this with the movie. The zip files are then uploaded. Please also correct the name to 'Movie EV#'.
The newly uploaded movies have the legend included

- Please remove the Movie and EV Table legends from the manuscript file.

We have removed these

- Source code legend and macro: Please provide reader access to the macro. Either include the entire macro in the methods or upload it as a text file (EV file type).

We have uploaded the macro as a txt file (macro.txt

- The manuscript sections are in the wrong order. Please have a look at our Guide to Authors for more information.
https://www.embopress.org/page/journal/14693178/authorguide#researcharticleguide<https://ddec1-0-en-

ctp.trendmicro.com:443/wis/clicktime/v1/query?url=https%3a%2f%2fwww.embopress.org%2fpage%2fjournal%2f14693178%2fauthorguide%23researcharticleguide&umid=f0c54d2a-344a-47cf-ac15-5e71402878ad&auth=8d3ccd473d52f326e51c0f75cb32c9541898e5d5-39044bc52c6c8ab41db5debec5c5bd42169b61af>
We have reordered the manuscript as per the guide

- I attach to this email a related manuscript file with comments by our data editors, mainly in the figure legends. Please address all comments and upload a revised file with tracked changes with your final manuscript submission.

We have made these changes. We have uploaded two manuscript versions – one with, and one without, the track changes shown.

- Finally, EMBO reports papers are accompanied online by A) a short (1-2 sentences) summary of the findings and their significance, B) 2-3 bullet points highlighting key results and C) a synopsis image that is 550x200-600 pixels large (width x height) in .png format. You can either show a model or key data in the synopsis image. Please note that the size is rather small and that text needs to be readable at the final size. Please send us this information along with the revised manuscript.
We have uploaded these in a separate file and also now include a figure.

We look forward to seeing a final version of your manuscript as soon as possible.

Kind regards,

Martina Rembold

Martina Rembold, PhD
Senior Editor
EMBO reports

****

Referee #2:

In my view the manuscript is interesting and would be useful to the field, although I think that a fuller explanation within the manuscript in response to the referees' comments (e.g. discussion of basal activation and how this affects the physiology of cells and embryos, demonstration of spatiotemporal functionality), would have improved the impact and novelty of the study. I have also since noticed another manuscript: Illes et al., 2021 PMID: 33718837. This seems more similar to the current tool's functionality than the Dowbaj publication (it activates 'optoYAP' by triggering nuclear translocation

and shows downstream functional changes, both in gene expression and in cell growth). This uses a different photoactivation mechanism to the current study.

2nd Revision - Editorial Decision 1st Jul 2022

Dr. Timothy Saunders
University of Warwick
Warwick Medical School
Warwick Medical School
Gibbett Hill Road
Coventry, West Midlands CV4 7LA
United Kingdom

Dear Tim,

Thank you for approving the last minor changes. I am now very pleased to accept your manuscript for publication in the next available issue of EMBO reports. Thank you for your contribution to our journal.

At the end of this email I include important information about how to proceed. Please ensure that you take the time to read the information and complete and return the necessary forms to allow us to publish your manuscript as quickly as possible.

As part of the EMBO publication's Transparent Editorial Process, EMBO reports publishes online a Review Process File to accompany accepted manuscripts. As you are aware, this File will be published in conjunction with your paper and will include the referee reports, your point-by-point response and all pertinent correspondence relating to the manuscript.

If you do NOT want this File to be published, please inform the editorial office within 2 days, if you have not done so already, otherwise the File will be published by default [contact: emboreports@embo.org]. If you do opt out, the Review Process File link will point to the following statement: "No Review Process File is available with this article, as the authors have chosen not to make the review process public in this case."

Should you be planning a Press Release on your article, please get in contact with emboreports@wiley.com as early as possible, in order to coordinate publication and release dates.

Thank you again for your contribution to EMBO reports and congratulations on a successful publication. Please consider us again in the future for your most exciting work.

Kind regards,

Martina

Martina Rembold, PhD
Senior Editor
EMBO reports

****

THINGS TO DO NOW:

Please note that you will be contacted by Wiley Author Services to complete licensing and payment information. The required 'Page Charges Authorization Form' is available here: https://www.embopress.org/pb-assets/embo-site/er_apc.pdf - please download and complete the form and return to embopressproduction@wiley.com

You will receive proofs by e-mail approximately 2-3 weeks after all relevant files have been sent to our Production Office; you should return your corrections within 2 days of receiving the proofs.

Please inform us if there is likely to be any difficulty in reaching you at the above address at that time. Failure to meet our deadlines may result in a delay of publication, or publication without your corrections.

All further communications concerning your paper should quote reference number EMBOR-2021-54401V3 and be addressed to emboreports@wiley.com.

Should you be planning a Press Release on your article, please get in contact with emboreports@wiley.com as early as possible, in order to coordinate publication and release dates.

EMBO Press Author Checklist

Corresponding Author Name: Timothy Saunders	
Journal Submitted to: EMBO Reports	
Manuscript Number: EMBOR-2021-54401V2	

USEFUL LINKS FOR COMPLETING THIS FORM
The EMBO Journal - Author Guidelines
EMBO Reports - Author Guidelines
Molecular Systems Biology - Author Guidelines
EMBO Molecular Medicine - Author Guidelines

Reporting Checklist for Life Science Articles (updated January 2022)
This checklist is adapted from Materials Design Analysis Reporting (MDAR) Checklist for Authors. MDAR establishes a minimum set of requirements in transparent reporting in the life sciences (see Statement of Task: 10.31222/osf.io/9sm4x). Please follow the journal's guidelines in preparing your manuscript.
Please note that a copy of this checklist will be published alongside your article.

Abridged guidelines for figures
1. Data
The data shown in figures should satisfy the following conditions:
- the data were obtained and processed according to the field's best practice and are presented to reflect the results of the experiments in an accurate and unbiased manner.
- ideally, figure panels should include only measurements that are directly comparable to each other and obtained with the same assay.
- plots include clearly labeled error bars for independent experiments and sample sizes. Unless justified, error bars should not be shown for technical replicates.
- if n<5, the individual data points from each experiment should be plotted. Any statistical test employed should be justified.
- Source Data should be included to report the data underlying figures according to the guidelines set out in the authorship guidelines on Data Presentation.

2. Captions
Each figure caption should contain the following information, for each panel where they are relevant:
- a specification of the experimental system investigated (eg cell line, species name).
- the assay(s) and method(s) used to carry out the reported observations and measurements.
- an explicit mention of the biological and chemical entity(ies) that are being measured.
- an explicit mention of the biological and chemical entity(ies) that are altered/varied/perturbed in a controlled manner.
- the exact sample size (n) for each experimental group/condition, given as a number, not a range;
- a description of the sample collection allowing the reader to understand whether the samples represent technical or biological replicates (including how many animals, litters, cultures, etc.).
- a statement of how many times the experiment shown was independently replicated in the laboratory.
- definitions of statistical methods and measures:
  - common tests, such as t-test (please specify whether paired vs. unpaired), simple χ2 tests, Wilcoxon and Mann-Whitney tests, can be unambiguously identified by name only, but more complex techniques should be described in the methods section;
  - are tests one-sided or two-sided?
  - are there adjustments for multiple comparisons?
  - exact statistical test results, e.g., P values = x but not P values < x;
  - definition of 'center values' as median or average;
  - definition of error bars as s.d. or s.e.m.

Please complete ALL of the questions below.
Select "Not Applicable" only when the requested information is not relevant for your study.

Materials

Newly Created Materials	Information included in the manuscript?	In which section is the information available? (Reagents and Tools Table, Materials and Methods, Figures, Data Availability Section)
New materials and reagents need to be available; do any restrictions apply?	Yes	Materials and Methods

Antibodies	Information included in the manuscript?	In which section is the information available? (Reagents and Tools Table, Materials and Methods, Figures, Data Availability Section)
For **antibodies** provide the following information:<br>- Commercial antibodies: RRID (if possible) or supplier name, catalogue number and or/clone number<br>- Non-commercial: RRID or citation	Yes	Table EV2

DNA and RNA sequences	Information included in the manuscript?	In which section is the information available? (Reagents and Tools Table, Materials and Methods, Figures, Data Availability Section)
**Short novel DNA or RNA including primers, probes**: provide the sequences.	Yes	Table EV1

Cell materials	Information included in the manuscript?	In which section is the information available? (Reagents and Tools Table, Materials and Methods, Figures, Data Availability Section)
**Cell lines:** Provide species information, strain. Provide accession number in repository **OR** supplier name, catalog number, clone number, and/**OR** RRID.	Yes	Materials and Methods
**Primary cultures:** Provide species, strain, sex of origin, genetic modification status.	Not Applicable	
Report if the cell lines were recently **authenticated** (e.g., by STR profiling) and tested for mycoplasma contamination.	Yes	All cell lines used verified with mycoplasma testing and STR profiling in the past 6 months

Experimental animals	Information included in the manuscript?	In which section is the information available? (Reagents and Tools Table, Materials and Methods, Figures, Data Availability Section)
**Laboratory animals or Model organisms:** Provide species, strain, sex, age, genetic modification status. Provide accession number in repository **OR** supplier name, catalog number, clone number, **OR** RRID.	Yes	Materials and Methods
**Animal observed in or captured from the field:** Provide species, sex, and age where possible.	Not Applicable	
Please detail **housing and husbandry conditions**.	Yes	Materials and Methods

Plants and microbes	Information included in the manuscript?	In which section is the information available? (Reagents and Tools Table, Materials and Methods, Figures, Data Availability Section)
**Plants:** provide species and strain, ecotype and cultivar where relevant, unique accession number if available, and source (including location for collected wild specimens).	Not Applicable	
**Microbes:** provide species and strain, unique accession number if available, and source.	Not Applicable	

Human research participants	Information included in the manuscript?	In which section is the information available? (Reagents and Tools Table, Materials and Methods, Figures, Data Availability Section)
If collected and within the bounds of privacy constraints report on age, sex and gender or ethnicity for all study participants.	Not Applicable	

Core facilities	Information included in the manuscript?	In which section is the information available? (Reagents and Tools Table, Materials and Methods, Figures, Data Availability Section)
If your work benefited from core facilities, was their service mentioned in the acknowledgments section?	Yes	Acknowledgements

Design

Study protocol	Information included in the manuscript?	In which section is the information available? (Reagents and Tools Table, Materials and Methods, Figures, Data Availability Section)
If study protocol has been **pre-registered, provide DOI in the manuscript**. For clinical trials, provide the trial registration number **OR** cite DOI.	Not Applicable	
Report the **clinical trial registration number** (at ClinicalTrials.gov or equivalent), where applicable.	Not Applicable	

Laboratory protocol	Information included in the manuscript?	In which section is the information available? (Reagents and Tools Table, Materials and Methods, Figures, Data Availability Section)
Provide DOI OR other citation details if **external detailed step-by-step protocols** are available.	Not Applicable	

Experimental study design and statistics	Information included in the manuscript?	In which section is the information available? (Reagents and Tools Table, Materials and Methods, Figures, Data Availability Section)
Include a statement about **sample size** estimate even if no statistical methods were used.	Not Applicable	
Were any steps taken to minimize the effects of subjective bias when allocating animals/samples to treatment (e.g. **randomization procedure**)? If yes, have they been described?	Not Applicable	
Include a statement about **blinding** even if no blinding was done.	Not Applicable	
Describe **inclusion/exclusion criteria** if samples or animals were excluded from the analysis. Were the criteria pre-established? If sample or data points were omitted from analysis, report if this was due to attrition or intentional exclusion and provide justification.	Not Applicable	
For every figure, are **statistical tests** justified as appropriate? Do the data meet the assumptions of the tests (e.g., normal distribution)? Describe any methods used to assess it. Is there an estimate of variation within each group of data? Is the variance similar between the groups that are being statistically compared?	Yes	Figure legends and Materials and Methods

Sample definition and in-laboratory replication	Information included in the manuscript?	In which section is the information available? (Reagents and Tools Table, Materials and Methods, Figures, Data Availability Section)
In the figure legends: state number of times the experiment was **replicated** in laboratory.	Yes	Figure legends
In the figure legends: define whether data describe **technical or biological replicates**.	Yes	Figure legends

Ethics

Ethics	Information included in the manuscript?	In which section is the information available? (Reagents and Tools Table, Materials and Methods, Figures, Data Availability Section)
Studies involving **human participants**: State details of **authority granting ethics approval** (IRB or equivalent committee(s), provide reference number for approval.	Not Applicable	
Studies involving **human participants**: Include a statement confirming that **informed consent** was obtained from all subjects and that the experiments conformed to the principles set out in the WMA Declaration of Helsinki and the Department of Health and Human Services Belmont Report.	Not Applicable	
Studies involving **human participants:** For publication of **patient photos**, include a statement confirming that consent to publish was obtained.	Not Applicable	
Studies involving experimental **animals**: State details of **authority granting ethics approval** (IRB or equivalent committee(s), provide reference number for approval. Include a statement of compliance with ethical regulations.	Yes	Materials and Methods
Studies involving **specimen and field samples:** State if relevant **permits** obtained, provide details of authority approving study; if none were required, explain why.	Not Applicable	

Dual Use Research of Concern (DURC)	Information included in the manuscript?	In which section is the information available? (Reagents and Tools Table, Materials and Methods, Figures, Data Availability Section)
Could your study fall under dual use research restrictions? Please check biosecurity documents and list of **select agents and toxins** (CDC): https://www.selectagents.gov/sat/list.htm	Not Applicable	
If you used a select agent, is the security level of the lab appropriate and reported in the manuscript?	Not Applicable	
If a study is subject to dual use research of concern regulations, is the name of the **authority granting approval and reference number** for the regulatory approval provided in the manuscript?	Not Applicable	

Reporting

The MDAR framework recommends adoption of discipline-specific guidelines, established and endorsed through community initiatives. Journals have their own policy about requiring specific guidelines and recommendations to complement MDAR.

Adherence to community standards	Information included in the manuscript?	In which section is the information available? (Reagents and Tools Table, Materials and Methods, Figures, Data Availability Section)
State if relevant guidelines or checklists (e.g., **ICMJE, MIBBI, ARRIVE, PRISMA**) have been followed or provided.	Yes	Author contributions
For **tumor marker prognostic studies**, we recommend that you follow the **REMARK** reporting guidelines (see link list at top right). See author guidelines, under 'Reporting Guidelines'. Please confirm you have followed these guidelines.	Not Applicable	
For **phase II and III randomized controlled trials**, please refer to the **CONSORT** flow diagram (see link list at top right) and submit the CONSORT checklist (see link list at top right) with your submission. See author guidelines, under 'Reporting Guidelines'. Please confirm you have submitted this list.	Not Applicable	

Data Availability

Data availability	Information included in the manuscript?	In which section is the information available? (Reagents and Tools Table, Materials and Methods, Figures, Data Availability Section)
Have **primary datasets** been deposited according to the journal's guidelines (see 'Data Deposition' section) and the respective accession numbers provided in the Data Availability Section?	Not Applicable	
Were **human clinical and genomic datasets** deposited in a public access-controlled repository in accordance to ethical obligations to the patients and to the applicable consent agreement?	Not Applicable	
Are **computational models** that are central and integral to a study available without restrictions in a machine-readable form? Were the relevant accession numbers or links provided?	Not Applicable	
If publicly available data were reused, provide the respective **data citations in the reference list**.	Not Applicable	
